# Validation of an online imitation-inhibition task

**DOI:** 10.3758/s13428-024-02557-3

**Published:** 2025-01-30

**Authors:** Mareike Westfal, Emiel Cracco, Jan Crusius, Oliver Genschow

**Affiliations:** 1 Leuphana University Lueneburg, Lueneburg, Germany; 2 Ghent University, Ghent, Belgium; 3 University of Greifswald, Greifswald, Germany

**Keywords:** Automatic imitation, Imitation-inhibition task, Online research, Survey software

## Abstract

People automatically imitate a wide range of different behaviors. One of the most commonly used measurement methods to assess imitative behavior is the imitation-inhibition task (Brass et al., 2000). A disadvantage of its original form is, however, that it was validated for laboratory settings—a time-consuming and costly procedure. Here, we present an approach for conducting the imitation-inhibition task in online settings. We programmed the online version of the imitation-inhibition task in JavaScript and implemented the task in online survey software (i.e., Qualtrics). We validated the task in four experiments. Experiment 1 (*N* = 88) showed that the typical automatic imitation effects can be detected with good psychometric properties. Going one step further, Experiment 2 (*N* = 182) directly compared the online version of the imitation-inhibition task with its laboratory version and demonstrated that the online version produces similar strong and reliable effects. In Experiments 3 and 4, we assessed typical moderator effects that were previously reported in laboratory settings: Experiment 3 (*N* = 93) demonstrated that automatic imitation can be reliably detected in online settings even when controlling for spatial compatibility. Experiment 4 (*N* = 104) found, in line with previous research, that individuals imitate hand movements executed by a robot less strongly than movements executed by a human. Taken together, the results show that the online version of the imitation-inhibition task offers an easy-to-use method that enables the measurement of automatic imitation with common online survey software tools in a reliable and valid fashion.

**Supplementary Information:**

The online version contains supplementary material available at 10.3758/s13428-024-02557-3.

## Introduction

People have an automatic tendency to imitate a variety of different behaviors, including simple movements (Brass et al., 2000; Genschow & Florack, 2014; Genschow & Schindler, 2016; Genschow et al., 2012), facial expressions (Dimberg, 1982), emotions (Dimberg, 1982; Hess & Fischer, 2013), and gestures (Bernieri, 1988; Cracco et al., 2018a, 2018b). Early research indicates that such imitative behavior fulfills an important function, as it fosters learning (e.g., Bandura, 1962). More recent research illustrates that imitation also fulfills a crucial social function, as it bonds humans more strongly together by creating feelings of affiliation (for a review, see Duffy & Chartrand, 2015).

A disadvantage of current tasks measuring imitative behavior is that they were validated for laboratory settings. Since the confidence crisis in psychological research (e.g., Open Science Collaboration, 2015), there is a call for large samples (e.g., Asendorpf et al., 2013), which makes conducting effortful laboratory experiments particularly challenging and costly. Moreover, laboratory experiments usually involve student samples, which has been criticized repeatedly (e.g., Henry, 2008; Stevens, 2011). One of the most commonly used tasks measuring imitative behavior is the imitation-inhibition task (Brass et al., 2000, 2001). Here we present a reliable and valid online version of this task and show that it can be implemented in common online survey software tools. This allows researchers to measure imitation flexibly via online platforms in different countries with diverse samples and cultures. Moreover, it facilitates the assessment of large sample sizes when high-powered studies are needed. Therefore, the online task is not only more time-efficient, but also generally more economical than its laboratory version.

## Automatic imitation and its underlying processes

The imitation-inhibition task is the most frequently used task to measure individuals’ automatic tendency to imitate on a trial-by-trial basis (Brass et al., 2000; for a meta-analysis, see Cracco et al., 2018a, 2018b). The imitation-inhibition task is regularly used in many different fields, including social (e.g., Cracco et al., 2018a, 2018b; De Souter et al., 2021; Genschow et al., 2022), cognitive (e.g., Brass et al., 2000; Cracco et al., 2015; Genschow et al., 2017), developmental (e.g., Simpson & Riggs, 2011), and neuro- (e.g., Darda & Ramsey, 2019; Hogeveen et al., 2015) and personality psychology (e.g., Hogeveen & Obhi, 2013; Obhi et al., 2013; Westfal et al., 2021). In the imitation-inhibition task, participants respond to two numerical cues with two different finger movements across multiple trials. Typically, they respond to the number “1” by lifting their index finger and to the number “2” by lifting their middle finger. At the same time as the number appears on the screen, participants see another person’s hand lifting the same finger (congruent trial), the other finger (incongruent trial), or no finger (neutral trial). The main dependent variable is reaction time, since this can be measured very reliably (e.g., Genschow et al., 2017). Error rates are also measured as a dependent variable although they are not always as reliably interpretable, since they depend heavily on participants making enough errors, which is not always the case (e.g., Cracco et al., 2018a, 2018b; Genschow et al., 2017).

The most commonly reported effect with the imitation-inhibition task is the congruency effect, which refers to faster reaction times and fewer error rates in congruent as compared to incongruent trials (for a meta-analysis, see Cracco et al., 2018a, 2018b). In addition, researchers can assess two other automatic imitation indices by implementing the neutral condition in which the hand on the screen does not move. Typically, participants respond faster and with fewer errors to congruent than to neutral trials (facilitation effect) and faster and with fewer errors to neutral than to incongruent trials (interference effect). Since the observation of a movement facilitates the execution of the same movement and inhibits the execution of another movement, it is generally agreed that the imitation-inhibition task measures automatic imitation (Cracco & Brass, 2019; Cracco et al., 2018a, 2018b; Heyes, 2011). Originally, the imitation-inhibition task was used in laboratory settings by measuring movement onsets with custom-built light sensors (e.g., Brass et al., 2000, 2001). More recent research demonstrates that the task produces similar reliable effects by measuring key release times on computer keyboards in the laboratory (e.g., Butler et al., 2015; Genschow et al., 2017)—a procedure that we adapted for use in the online environment.

In principle, three different components may generally contribute to the typical automatic imitation effects: movement compatibility (e.g., observing a lifting movement while executing a lifting a movement), effector compatibility (e.g., observing the index finger while executing an index finger movement), and spatial compatibility (e.g., observing an effector or a movement on the left side of space while executing a movement on the left side of space). To control for spatial compatibility, past research used different methods (cf., Cracco et al., 2018a, 2018b). In the most commonly used method, researchers rotate the presented hand stimuli by 90°. By doing so, the observed finger movements are no longer in the same spatial location as the executed finger movements (e.g., Press et al., 2005). Another method that has been used is to present both left hands and right hands to the participants (e.g., Catmur & Heyes, 2011). Past research found robust effector and movement compatibility effects even when controlling for spatial compatibility (for a meta-analysis, see Cracco et al., 2018a, 2018b) further supporting the idea that the imitation-inhibition task is a measure of automatic imitation.

While there is consensus in the literature that the imitation-inhibition task measures imitative behavior (Cracco & Brass, 2019), there is currently a debate as to the degree to which social factors can modulate or manipulate automatic imitation. On the one hand, for example, there is consistent evidence for the idea that focusing on others, as compared to the self (e.g., Genschow et al., 2019; Spengler et al., 2010), or observing human as compared to non-human actions (e.g., Bird et al., 2007; Kilner et al., 2003; Klapper et al., 2014; Press et al., 2005), increases automatic imitation. On the other hand, due to several failed replications, the evidence for the idea that ingroup versus outgroup members (e.g., De Souter et al., 2021; Genschow & Schindler, 2016; Genschow et al., 2023; Gleibs et al., 2016; Rauchbauer et al., 2016) or direct versus averted gaze (e.g., Trilla et al., 2020; Wang et al., 2010) increases automatic imitation is rather unclear. One possible reason for the inconclusive evidence is that the effect size of social modulation is rather small (Cracco et al., 2018a, 2018b), which calls for a method that makes it possible to administer the imitation-inhibition task within large samples. The online version of the imitation-inhibition task we present here can fill this gap by facilitating data collection of large and diverse samples.

## Administering the imitation-inhibition task online

As data collection in laboratory experiments is usually time-consuming, effortful, and mainly limited to university samples (e.g., Thomas, 2011), making it very difficult to conduct studies across different countries and cultures (Hanel & Vione, 2016), a steadily increasing number of psychological studies nowadays take place online (e.g., Buhrmester et al., 2018). Indeed, with the help of different survey platforms (e.g., Qualtrics; www.qualtrics.com), it is now easy to implement high-powered studies online even with little programming knowledge. However, while online surveys can be easily used to assess simple questionnaires, there exist few solutions for assessing behavior, such as automatic imitation for instance, in online settings. In principle, one could measure imitative behavior by using webcams, which are available on some platforms, and use specific video software to track participants’ motions. However, such solutions are somewhat complicated and effortful to implement. In addition, the quality of the data depends on a variety of technical details such as the angle of the webcam and the resolution of the produced videos. Moreover, videotaping participants requires special ethical approval and data protection regulations, which may complicate running experiments involving webcams. Since previous research conducted in the laboratory has already successfully measured automatic imitation from participants’ key releases (e.g., Butler et al., 2015; Genschow et al., 2017), an easier-to-implement solution than motion tracking is to measure automatic imitation by letting participants release keys on their computer keyboard in response to finger lifting movements presented on the screen.

However, there are several solutions for creating response time tasks using online construction software solutions that include integrated toolkits (e.g., Labvanced, https://www.labvanced.com/; Inquisit, https://www.millisecond.com/). Similarly, there are also downloadable tools for creating online experiments with graphical user interfaces (e.g., PsychoPy, https://www.psychopy.org/). Still, one disadvantage of these software solutions is that the integrated online toolkits do not offer a complete range of functions needed for some response time tasks. For example, they do not offer the possibility of a key release measurement method, which is necessary for the imitation-inhibition task. In addition, the experiments created often cannot be run directly online but require an additional platform (e.g., PsychoPy, https://www.psychopy.org/ and Pavlovia, https://pavlovia.org/). Moreover, participants might have to download a program or file when they participate in a study, which many participants shy away from. A solution for these problems is to program reaction-time-based measures from scratch with other tools, such as JavaScript libraries (e.g., jsPsych, www.jspsych.org; de Leeuw, 2015; de Leeuw & Motz, 2016; Hilbig, 2016; Pinet et al., 2017). However, without programming knowledge, the implementation of such measures can be a time-consuming and a challenging hurdle that not every researcher is willing and able to overcome. Thus, what is needed is a validated version of the imitation-inhibition task that is programmed (e.g., by using the jsPsych library) in such a way that it can be easily implemented on a personal server or a simple survey platform (i.e., Qualtrics)—an approach we implemented in this article. This way, researchers and students interested in assessing automatic imitation do not have to program the task themselves, but can make use of the validated version we present here even if they have limited programming knowledge.

## Construction of the online version of the imitation-inhibition task

To program the online version of the imitation-inhibition task, we used the JavaScript library jsPsych (de Leeuw, 2015) by applying existing plugins and generating new plugins (i.e., jspsych-check-response, jspsych-image-keyboard-release and, jspsych-fixation-image-keyboard-release; Cracco, 2020) to custom-build the task. We have uploaded the whole task, in addition to a list of all plugins we used and developed, to the Open Science Framework (OSF; https://osf.io/q7fju/) to make the programming process transparent. After creating the code, we modified it so that it can be used in Qualtrics. The OSF folder also contains detailed instructions and a tutorial for this purpose. In addition, we uploaded to the OSF folder analysis scripts to preprocess and analyze the gathered data. When using all the tools we provide on OSF, researchers without much programming knowledge or with little time available can very easily and quickly apply and analyze the online version of the imitation-inhibition task.

### The procedure for the online version of the imitation-inhibition task

In line with previous research conducted in the laboratory (Butler et al., 2015; Genschow et al., 2017), the online version of the imitation-inhibition task measures response latencies and error rates by detecting key releases on participants’ computer keyboards. Thus, to complete the online imitation-inhibition task, participants need a computer with a keyboard. They will not be able to participate on a tablet without keyboard or on a cell phone. We advise researchers to mention this information when recruiting participants. The reason for this restriction is that (1) stimulus movements may not be sufficiently recognizable on small devices, and (2) mobile devices could be used with potential distractors in the surroundings (e.g., traffic). Limiting the task to keyboards eliminates such situations, increasing the data quality.

Although the online version of the imitation-inhibition task is similar to the original laboratory-based task developed by Brass et al., (2000, 2001), we added a new practice procedure with two consecutive practice phases, because in online studies, participants cannot ask the experimenter for clarification and often read the instructions poorly (or not at all), which increases the risk of misunderstanding the instructions (e.g., Birnbaum, 2004; De Man et al., 2021; Reips, 2000). Additionally, in reaction time tasks, it is more common to press a key and not to hold and release it, so in the online setting in particular, it was important to us that participants had a clear understanding of the task procedure. To start the task, participants have to click a button in the center of the screen to switch to full-screen mode. This reduces the risk that participants become distracted by other programs and browser tabs that might be open while running through the task. Afterwards, participants receive general instructions about the experiment and the imitation-inhibition task. Crucially, participants are instructed to respond with their right index finger and middle finger only. To illustrate the general instructions, participants see a gif of a participant performing the task correctly. Next, participants complete two practice phases.

In the first practice phase, participants are instructed to first press and hold down the “g” key with their right index finger and the “h” key with their right middle finger. As soon as they press down both keys, the participants see a fixation cross for 500 ms, followed by a picture of the number cue “1” or “2”. Within a time window of 2000 ms, participants need to respond as fast as possible to the number cue “1” by lifting their index finger and to the number cue “2” by lifting their middle finger. After lifting their finger, participants receive accuracy feedback (i.e., CORRECT in green or WRONG in red letters) displayed for 1000 ms. If participants do not lift any finger, they receive an instruction to lift a finger. Then, a post-trial gap of 950 ms follows until the next trial starts again. After 10 trials, participants receive feedback about their overall performance. If a participant commits more than two errors, they have to repeat the practice phase until they reach the threshold of at least eight correct trials.

**Fig. 1:**
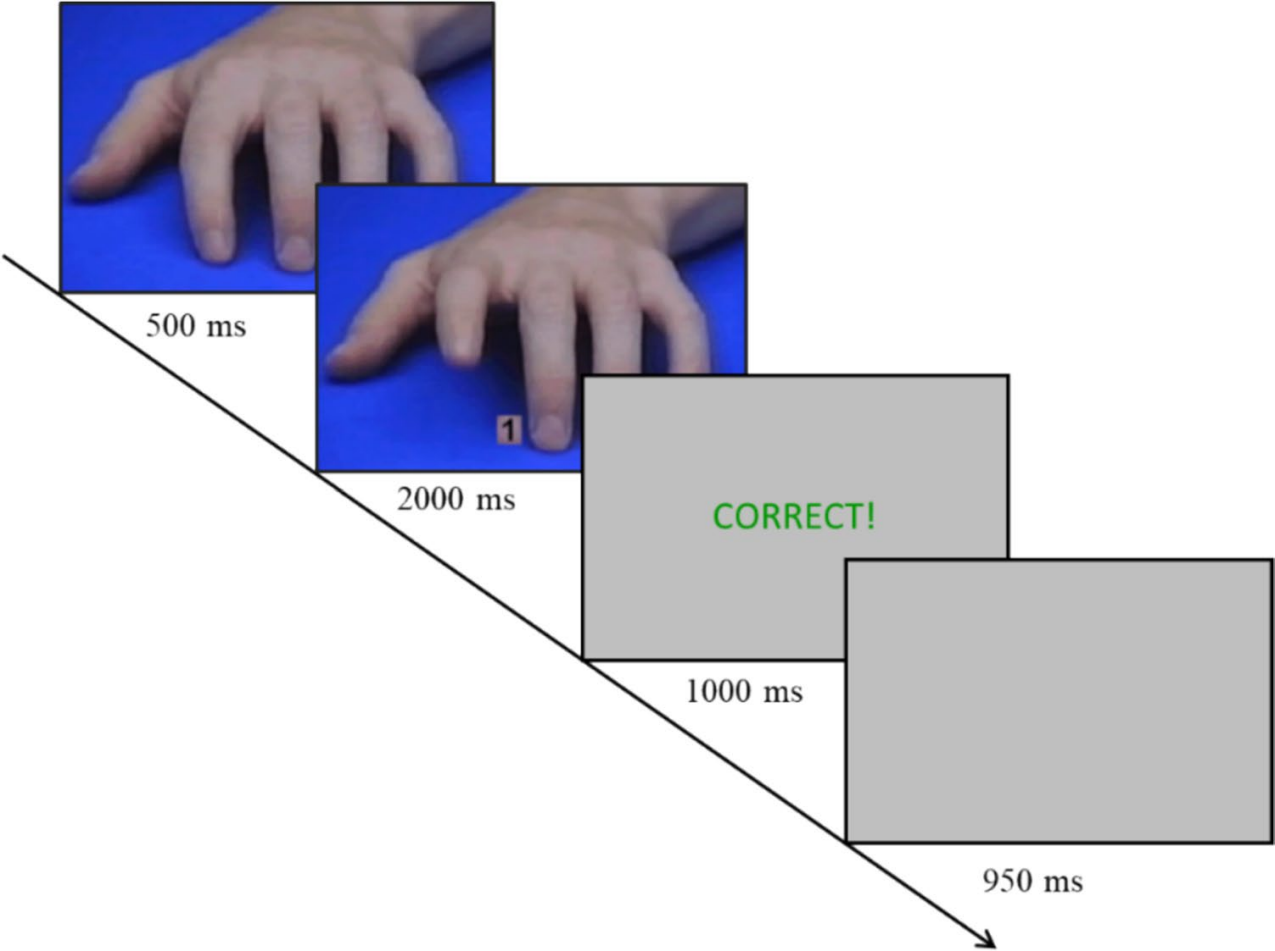
Schematic representation of a congruent practice trial

The procedure in the second practice phase consists of 12 trials and is similar to the procedure in the first practice phase (see Fig. 1). In contrast to the first practice phase, though, the number cues are now combined with images of another person’s hand. Specifically, instead of the fixation cross, another person’s hand in resting position is shown for 500 ms. Afterwards, a picture of the same hand lifting either the index or the middle finger with the number cues presented between the index and middle finger is shown for a maximum of 2000 ms. The participants are again instructed to react to the number cue as fast as possible by lifting their fingers as before. Participants receive accuracy feedback exactly as in the first practice phase. To ensure that participants understand the task, they have to repeat this practice phase until they commit fewer than four errors.

After the two practice phases, participants start with the experimental phase. The procedure in a typical experimental block is the same as the second practice block, except that participants receive no accuracy feedback. Between each block, participants can take a self-paced break. In addition to the pictures with the model raising the middle finger or the index finger, researchers can implement a neutral condition in which they present pictures of a model not raising a finger. This way, pictures of three different trial types can be shown: In congruent trials, participants are instructed to respond with the same finger as the model. In incongruent trials, participants are instructed to respond with another finger. In neutral trials, participants respond to the cue with a finger while the model’s hand does not move at all.

## A survey-software solution for the imitation-inhibition task

The task can be directly implemented on one’s own (or any other) server by using jsPsych software. On the Open Science Framework (OSF; https://osf.io/q7fju/), we provide a detailed tutorial on how to implement the task on a server. In addition, we programmed a version that can be implemented in online survey platforms. Here, we present a solution to implement the imitation-inhibition task in one of the most widely used online survey platforms— Qualtrics. Although we focus here on instructions for the implementation of the task within Qualtrics or on a server, with a slight adjustment, the task should theoretically also be implementable on other server providers such as MindProbe (https://mindprobe.eu/), Cognition.run (https://www.cognition.run/), Pavlovia (www.pavlovia.org), or other survey platforms that support JavaScript add-ins.

In Qualtrics, the task is implemented by using HTML and JavaScript code to a “text entry” survey question, so that a second window overrides the actual question and shows the task. We created different.qsf files for different purposes: the whole original task (with congruent, incongruent, and neutral trial conditions), the original task without a neutral condition, a version of the original task with pictures controlled for spatial compatibility (rotated by 90°) with and without a neutral condition, the task assessing imitative and spatial compatibility separately, a basic version of the task that can contain a picture above the hand stimuli (again with and without a neutral condition), and a version of the task in which two different hand conditions can be implemented (e.g., robotic vs. human hand). Furthermore, we created two versions of the basic original task.qsf files (one with and one without neutral trials) that contain the practice phase and the experimental blocks separated and independently of each other in individual “text entry” questions without instructions, to make the task even more customizable. We have uploaded the different task versions as.qsf files to our OSF folder along with a detailed tutorial (i.e., “Tutorial—Qualtrics implementation”) that describes each task version and its implementation step-by-step. Here, we provide further important information for the implementation and use of the online version of the imitation-inhibition task on Qualtrics.

**Which content of the code should be added?** Our downloadable Qualtrics-.qsf files can be used to automatically create a survey that already contains the task. Contents of the code itself can also be changed by code-savvy persons in Qualtrics by clicking " < / > JavaScript" under question behavior of the “text entry” questions that contain the task. Everything is explained in more detail in the tutorial on the OSF.

**How are reaction times measured?** Reaction times are measured using the provided and adapted plugin from jsPsych (de Leeuw, 2015). With this plugin, reaction times are recorded following de Leeuw (2015) by recording timestamps when the stimulus appears and when the subject responds (i.e., releases the button) and storing the differences in milliseconds. The reaction times that are recorded are very accurate, reliable, and already validated in previous experiments (e.g., Pinet et al., 2017). For example, de Leeuw and Motz (2016) showed that although JavaScript exhibits a small delay, JavaScript using jsPsych is just as sensitive to reaction times between conditions as any laboratory platform. Similar results were presented by Hilbig (2016). After the reaction times of the individual trials are recorded, they are stored block-wise as.csv files in the respective embedded variables and can be read out later individually. In this way, no trial is lost, and trial-wise exclusion criteria can be applied.

**How exactly is the data stored?** We store the trial-wise data per block individually in comma-separated.csv files. The script is written to store up to 10 blocks of the imitation-inhibition task. Theoretically, more than 10 blocks can be stored, but this would require adapting the script and adding new embedded variables to the survey flow in Qualtrics. Since we could not create an infinite number of storage embedded variables, we decided to limit the script to 10 variables. Above that it would be too exhausting for the participants anyway to further participate in such a reaction time task. Since the trial number per block can also be adjusted, 10 blocks should be sufficient. This block-wise trial data is stored in a single embedded variable for each block per participant and can be read and processed individually later in R (R Core Team, 2022). In addition to the code of the imitation-inhibition task, on OSF, we provide detailed technical instructions and codes on how to implement the task, how to preprocess the data, and how to analyze it.

**How are pictures handled?** The pictures are read in and loaded automatically into the computer’s cache before the practice phases trials start, so they are directly available and do not interfere with the reaction times. Theoretically, images can be used other than the ones we used during the validation experiments. Our images all have a size of 400 × 267 pixels, but images of a different size can also be used.

**Fig. 2:**
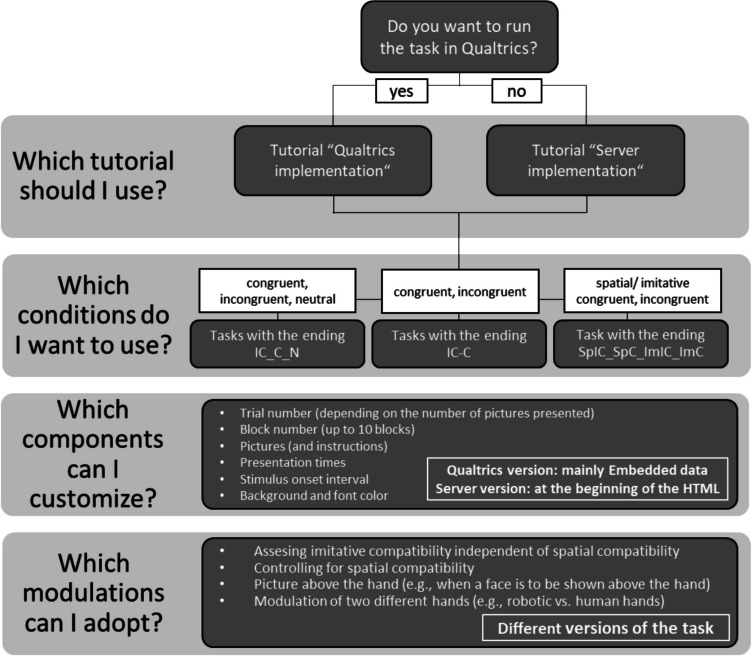
Flowchart of the customization possibilities with the online imitation-inhibition task

**How can the individual parts of the experiment be customized?** Researchers can adjust the task in a number of ways if they wish. For example, the pictures of the practice phases and the experimental phase can be exchanged with any other picture. The default are the pictures we used in the validation experiments. Likewise, custom pictures can be inserted as long as they are on an appropriately accessible location (e.g., GitHub, an accessible cloud, or personal server). Moreover, we provide a number of variables in the survey flow that can be customized. In particular, researchers can vary the timing (e.g., of the fixation, trial duration, and post-trial gap), the block count, and the number of stimulus repetitions within one block (i.e., the trial count within one block), as well as the color of the background or the font of the task. All of these settings can be conveniently changed in Qualtrics itself without having to open the task in the JavaScript window of Qualtrics. For this, we use the "Embedded data" feature provided by Qualtrics (i.e., a facility provided by Qualtrics to easily add or save data; https://www.qualtrics.com/support/survey-platform/survey-module/survey-flow/standard-elements/embedded-data/). A detailed tutorial explaining how these features can be changed is available on our OSF. Figure 2 shows the possibilities for customization and leads to the different tutorials that contain more information about the customization handling.

The flow of the task and the two practice phases cannot be changed directly. For this, an interested researcher would have to adapt the JavaScript code. Likewise, the instructions of the task cannot be changed directly in Qualtrics Embedded data variables. However, the places where the instructions can be changed are marked accordingly in the JavaScript code and are placed so that they are easily found. To aid these kinds of changes, we added short remarks in the tutorial in the OSF. Moreover, as mentioned, we provide second.qsf files for the basic tasks in which only the two practice phases in one "text entry" question and ten single blocks of the task in other "text entry" questions are available without instructions or breaks. Using this.qsf file or its code, researchers can create their own imitation-inhibition task with individual instructions or break messages. By changing the instructions, using the modulated tasks, or using the task with the separated blocks, the task can be framed in various ways allowing, for example, top-down manipulations to be easily implemented.

**How can the data of the task be preprocessed and analyzed?** On OSF (https://osf.io/q7fju/), we provide R scripts (https://cran.r-project.org/) to preprocess and analyze the data. Theoretically, no knowledge of R is necessary for this approach.

**Can I try the task?** Yes, the task is available as a demo version at the following URL: https://www.automatic-imitation.com/. This is a basic version of the task including all three conditions (congruent, incongruent, and neutral).

## Empirical validation

To validate the online version of the imitation-inhibition task, we conducted four experiments. In Experiments 1 and 2, we tested the task for its functionality and compared it with a laboratory sample in terms of reliability and effect size. In Experiment 3, we tested whether the task produces the expected findings when controlling for spatial compatibility. Finally, in Experiment 4, we investigated whether one of the most frequently reported social moderators—bottom-up animacy—influences automatic imitation measured with the online version of the imitation-inhibition task.

## Experiment 1

In Experiment 1, we tested whether the online version of the imitation-inhibition task reliably produces the expected effects (i.e., congruency effect, facilitation effect, interference effect). The experiment was preregistered at AsPredicted (https://aspredicted.org/k9zz-p62v.pdf).

### Method

#### Participants 

We aimed to detect a medium to small effect size of *d* = 0.32, which is well below the average effect sizes found in the laboratory (Cracco et al., 2018a, 2018b), as we wanted to be sure to find the effect in the online environment. With power of β = 0.90, 86 participants were needed to detect such an effect. To compensate for potential dropouts based on our preregistered exclusion criteria, we preregistered a sample of 100 participants. We recruited participants via Amazon’s Mechanical Turk in return for compensation of $1.00. Only MTurkers located in the United States with a Hit Approval Rate over 85% were invited to participate in the study. Participants could only take part in the study with a laptop or computer. In line with our preregistration, data from a total of 12 participants were excluded from data analysis because they had less than 33 trials in one or more of the conditions (*n* = 7), or indicated that they did not use the right hand during the experimental blocks (*n* = 5). The final sample consisted of 88 participants (40 female, 48 male) with age ranging from 20 to 64 years (*M* = 35.41, *SD* = 10.62). Fourteen participants were left-handed and 74 participants were right-handed.

#### Procedure 

The procedure was exactly the same as described above. The experiment consisted of two practice blocks with accuracy feedback to familiarize participants with the task. As described above, participants had to repeat the first practice phase until they reached the threshold of 8 out of 10 accurate trials, and they had to repeat the second practice phase until they reached the threshold 8 out of 12 accurate trials. Two participants had to repeat the first practice block, 41 participants had to repeat the second practice block, and seven participants had to repeat both practice blocks. Afterwards, participants ran through five experimental blocks without receiving feedback. Per block, we presented 30 trials in random order. Participants could take a self-paced break between the experimental blocks. In total, the experiment consisted of 150 trials (50 incongruent, 50 congruent, and 50 neutral trials). To prepare the data for analysis, we removed extremely fast and slow reaction times in line with our preregistration. That is, we removed trials with reaction times below 100 ms (0.46%) and latencies below (0.02%) or above (0.93%) 3 *SD*s of a participant’s mean. For the analyses of the latencies, we removed erroneous trials as well (7.11%).

**Fig. 3:**
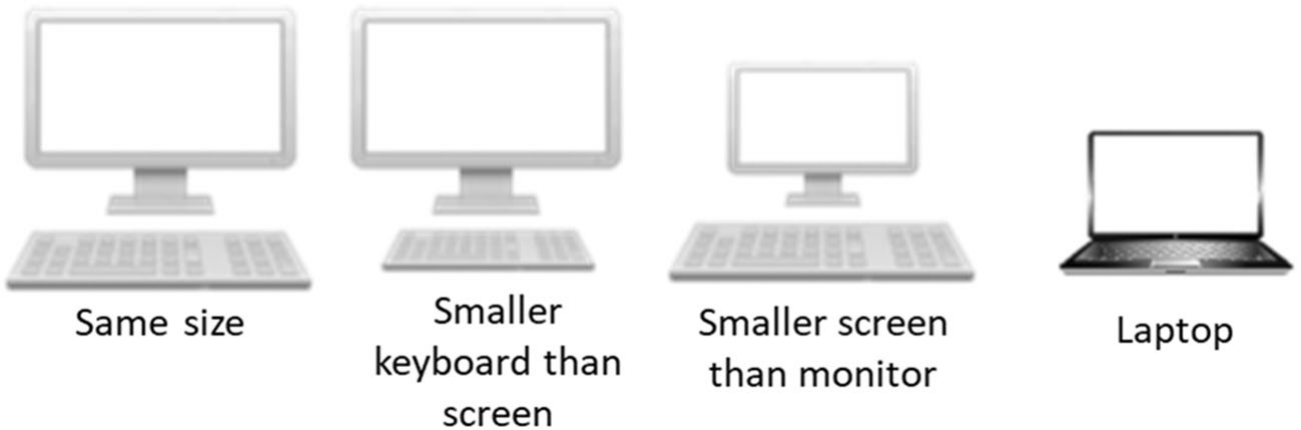
Stimuli for estimation of the ratio between keyboard and screen

At the end, participants indicated demographic data (i.e., gender and age) and which hand they used during the experimental blocks (left hand, right hand, or both hands). In addition, participants estimated the ratio between their keyboard and their screen (same size, smaller keyboard than screen, smaller screen than monitor, and laptop; see Fig. 3), indicated what kind of keyboard they had used (i.e., regular keyboard with prominent keys, regular keyboard with flat keys, Mac keyboard with prominent keys, and Mac keyboard with flat keys), whether they used a computer with an external monitor or a laptop, and their handedness (right- or left-handed). Additionally, we extracted from the User-Agent string which browser the participants were using. On average, participants needed *M* = 12.37 (*SD* = 5.04) minutes to complete the task. The task can be found, together with the stimuli, plugins, and further material used, on the OSF (https://osf.io/q7fju/).

### Results

#### Latencies 

**Fig. 4:**
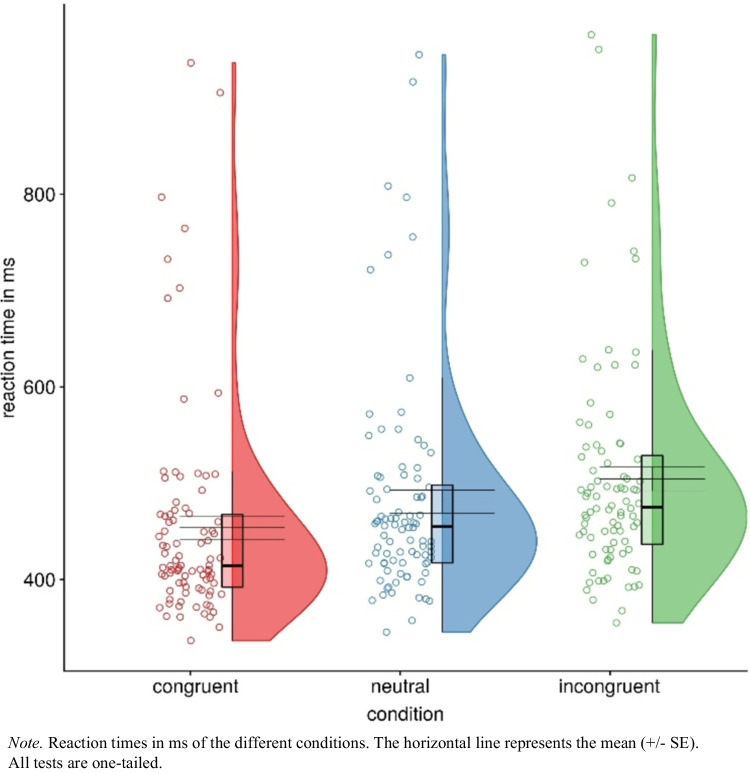
Response times in Experiment 1

For the response times, we applied three preregistered *t*-tests for dependent samples to test for the presence of the typical imitation-inhibition effects (see Fig. 4). With respect to the congruency effect, the results indicate that participants responded faster in congruent trials (*M* = 453.77 ms, *SD* = 113.44), than in incongruent trials (*M* = 504.50 ms, *SD* = 114.88), *t*(87) = 13.24, *p* < 0.001, *d*_*z*_ = 1.41, CI 95% [1.11, 1.71], η_p_^2^ = 0.67. Also, the facilitation effect was significant: participants responded faster in congruent trials (*M* = 453.77 ms, *SD* = 113.44) than in neutral trials (*M* = 480.85 ms, *SD* = 112.91), *t*(87) = 12.09, *p* < 0.001, *d*_*z*_ = 1.29, CI 95% [1.00, 1.57], η_p_^2^ = 0.63. Finally, with respect to the interference effect, participants responded faster in neutral trials (*M* = 480.85 ms, *SD* = 112.91) than in incongruent trials (*M* = 504.50 ms, *SD* = 114.88), *t*(87) = 8.89, *p* < 0.001, *d*_*z*_ = 0.95, CI 95% [0.69, 1.20], η_p_^2^ = 0.48.

#### Error rates 

**Fig. 5:**
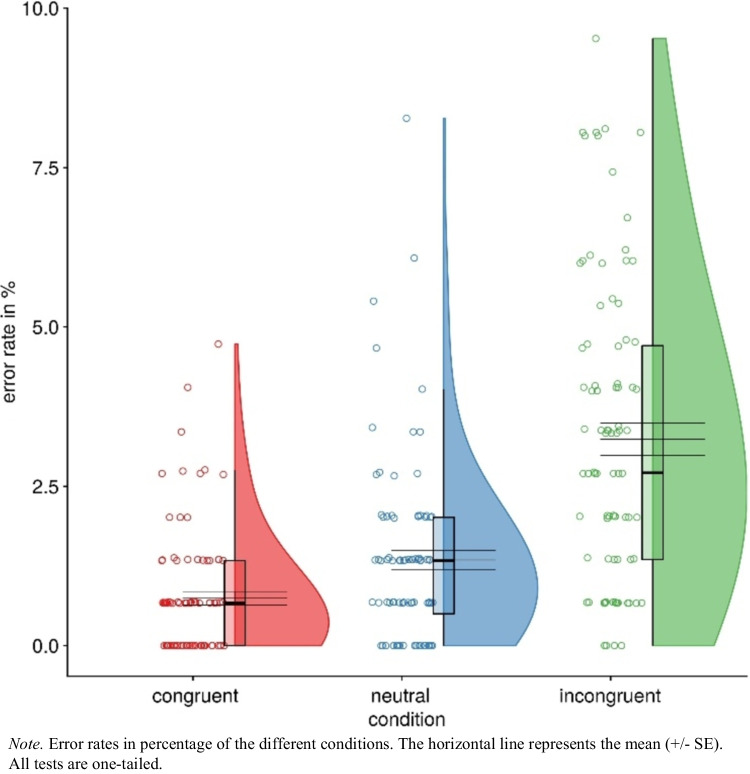
Error rates of Experiment 1

In a second step, we ran the same analyses for the error rates.^1^ In line with the latencies, we detected a congruency effect as participants made fewer errors in congruent trials (*M* = 0.74%, *SD* = 0.98) than in incongruent trials (*M* = 3.24%, *SD* = 2.38), *t*(87) = 9.89, *p* < 0.001, *d*_*z*_ = 1.05, CI 95% [0.79, 1.31], η_p_^2^ = 0.53. Moreover, we found a facilitation effect showing that participants committed fewer errors in congruent trials (*M* = 0.74%, *SD* = 0.98), as compared to neutral trials (*M* = 1.34%, *SD* = 1.44), *t*(87) = 4.55, *p* < 0.001, *d*_*z*_ = 0.49, CI 95% = [0.26, 0.70], η_p_^2^ = 0.20. Finally, we found an interference effect as well: In neutral trials, participants made fewer errors (*M* = 1.34%, *SD* = 1.44) than in incongruent trials (*M* = 3.24%, *SD* = 2.38), *t*(87) = 8.29, *p* < 0.001, *d*_*z*_ = 0.88, CI 95% = [0.64, 1.13], η_p_^2^ = 0.44. The error rates of the effects are shown in Fig. 5.

#### Reliability

To investigate the reliability of the task, we calculated split-half reliabilities of the respective effects on the basis of odd and even trials using the Spearman–Brown coefficient in line with previous research on the imitation-inhibition task (Genschow et al., 2017). For the latencies, the congruency effect achieved a reliability of ρ^*^ = 0.72, the facilitation effect a reliability of ρ^*^ = 0.38, and the interference effect a split-half reliability of ρ^*^ = 0.49. For the error rates, the congruency effect achieved a reliability of ρ^*^ = 0.64, the facilitation effect a reliability of ρ^*^ = 0.45, and the interference effect a split-half reliability of ρ^*^ = 0.42. The reliabilities of the effects of the task are comparable to the reliability reported for laboratory experiments in prior research (Genschow et al., 2017).

#### Explorative analyses 

In additional analyses, we tested whether the ratio between participants’ keyboard and their screen, the keyboard they had used, the browser they had used, their handedness, whether they used an external monitor or a laptop, and the number of repetitions in the first and second practice block influenced the imitation-inhibition indices. None of these factors affected any of the three automatic imitation indices for latencies, *F*s < 2.25, *p*s > 0.145 or for error rates, *F*s < 3.94, *p*s > 0.050.

### Discussion

By applying the online version of the imitation-inhibition task, we replicated the typical imitation-inhibition effects for latencies as well as error rates. The results of the task were not influenced by the ratio between participants’ keyboard and their screen, the computer keyboard and browser used, their handedness, or the number of repetitions in one of the practice blocks. This illustrates the robustness of the task. Moreover, the effects of the task in terms of effect size and reliability were in the same range as reported for laboratory experiments in prior research (e.g., Cracco et al., 2018a, 2018b; Genschow et al., 2017). However, to actually compare the online version of the imitation-inhibition task with its laboratory equivalent, an experiment with both tasks is needed—an approach we followed in Experiment 2.

## Experiment 2

In Experiment 2, we empirically compared the imitation-inhibition task in an online setting with a laboratory setting and tested whether one of the tasks produces stronger and more reliable effects. The experiment was preregistered at AsPredicted (https://aspredicted.org/775h-8h4y.pdf).

### Method

#### Participants 

Applying the same power analysis as for Experiment 1, we collected an online sample of 100 participants. We recruited the participants via Amazon’s Mechanical Turk in return for compensation of $1.00. The Hit Approval Rate and the location was the same as in Experiment 1. Participants could again only take part in the study with a laptop or computer. In line with our preregistration, data from a total of 16 participants were excluded from data analysis because they had less than 33 valid trials in one or more of the conditions (*n* = 13) or did not use the right hand during the experimental blocks (*n* = 4). One person met both exclusion criteria. The final online sample consisted of 84 participants (40 female, 42 male, 2 other) with age ranging from 21 to 73 years (*M* = 35.74, *SD* = 12.07). Eight participants were left-handed and 76 participants were right-handed. To ensure that the data collection for both samples (i.e., online and laboratory) was similar, we recruited the participants for the online experiment on several different days spread over 2 weeks to match the days on which we assessed the participants in the laboratory.

We collected the same number of participants in the laboratory as we did online to obtain a balanced sample. Specifically, for the laboratory sample we recruited 100 participants on the campus of the university of Cologne (Germany) in return for compensation of a chocolate bar or a coffee. In line with our preregistration, data from two participants were excluded from data analysis because they had less than 33 valid trials in one or more of the conditions. The final laboratory sample consisted of 98 participants (46 female, 52 male) with age ranging from 16 to 55 years (*M* = 23.42, *SD* = 5.51). Fourteen participants were left-handed and 84 participants were right-handed.

#### Procedure 

We used the exact same procedure as in Experiment 1 for both the online and the laboratory sample. To prepare the data for analysis, as preregistered, we removed trials with reaction times below 100 ms for the latency and error rate analyses (online sample: 1.11%; laboratory sample: 0.07%). We also removed latencies below (online sample: 0.03%; laboratory sample: 0%) and above (online sample: 0.97%; laboratory sample: 0.91%) 3 *SD*s of a participant’s mean. For the analyses of the latencies, we discarded erroneous trials as well (online sample: 8.09%; laboratory sample: 5.43%).

### Results

#### Latencies

We completed several preregistered tests. First, we tested for the presence of the typical imitation-inhibition effects. In line with Experiment 1, the typical imitation effects were significant in both samples. In the online sample, participants responded faster in congruent trials (*M* = 463.26 ms, *SD* = 104.51) than in incongruent trials (*M* = 522.95 ms, *SD* = 111.09), *t*(83) = 14.32, *p* < 0.001, *d*_*z*_ = 1.56, CI 95% [1.24, 1.88], η_p_^2^ = 0.71. Also, the facilitation effect reached significance: The participants responded faster in congruent (*M* = 463.26 ms, *SD* = 104.51) than in neutral trials (*M* = 494.57 ms, *SD* = 104.10), *t*(83) = 13.43, *p* < 0.001, *d*_*z*_ = 1.47, CI 95% = [1.15, 1.77], η_p_^2^ = 0.69. Finally, we found a significant interference effect as participants responded faster to neutral trials (*M* = 494.57 ms, *SD* = 104.10) than to incongruent trials (*M* = 522.95 ms, *SD* = 111.09), *t*(83) = 9.98, *p* < 0.001, *d*_*z*_ = 1.09, CI 95% = [0.82, 1.36], η_p_^2^ = 0.55.

In the laboratory sample, we found the same effects. Participants responded faster in congruent trials (*M* = 416.36 ms, *SD* = 46.36) than in incongruent trials (*M* = 468.21 ms, *SD* = 60.37), *t*(97) = 15.50, *p* < 0.001, *d*_*z*_ = 1.57, CI 95% [1.27, 1.86], η_p_^2^ = 0.71. They responded faster in congruent trials (*M* = 416.36 ms, *SD* = 46.36) than in neutral trials (*M* = 442.29 ms, *SD* = 50,18), *t*(97) = 13.11, *p* < 0.001, *d*_*z*_ = 1.32, CI 95% = [1.05, 1.59], η_p_^2^ = 0.64, and faster to neutral trials (*M* = 442.29 ms, *SD* = 50,18) than to incongruent trials (*M* = 468.21 ms, *SD* = 60.37), *t*(97) = 10.22, *p* < 0.001, *d*_*z*_ = 1.03, CI 95% = [0.78, 1.27], η_p_^2^ = 0.52.

**Fig. 6:**
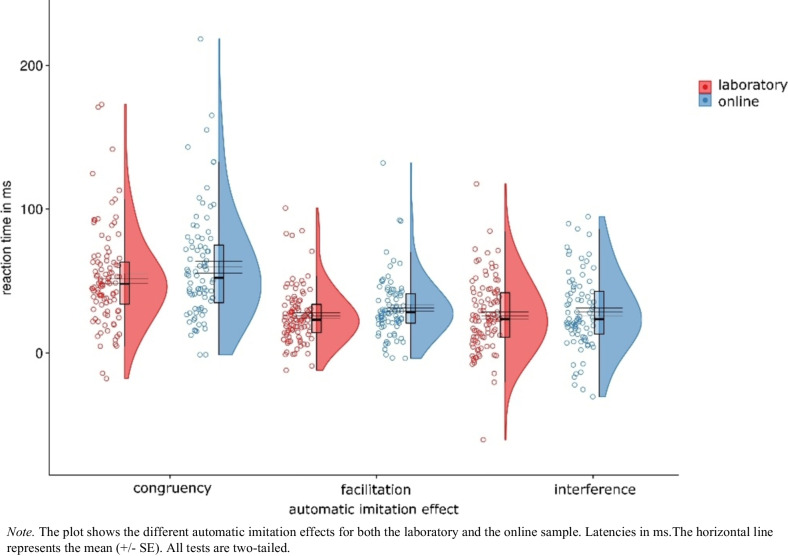
Reaction time comparison between online and laboratory samples

Moreover, in line with previous research on automatic imitation (for a review, see Cracco et al., 2018a, 2018b) we compared the automatic imitation effects between the laboratory and the online sample directly. For this purpose, we first calculated the individual automatic imitation effects per participant. For the congruency effect, the congruent trial condition mean values were subtracted from the incongruent mean values for each participant individually; for the facilitation effect the neutral mean values were subtracted from the congruent trial condition mean values; and for the interference effect the neutral mean values were subtracted from the incongruent trial condition mean values. The direct comparison of the typical imitation-inhibition effects from the online and laboratory samples showed no meaningful differences between the samples in terms of the congruency effect (laboratory: *M* = 51.85 ms, *SD* = 33.11; online: *M* = 59.70 ms, *SD* = 38.21), *t*(180) = 1.48, *p* = 0.140, *d*_*z*_ = 0.22, CI 95% = [− 0.07, 0.51], η_p_^2^ = 0.05, the facilitation effect (laboratory: *M* = 25.92 ms, *SD* = 19.58; online: *M* = 31.31 ms, *SD* = 21.37), *t*(180) = 1.78, *p* = 0.078, *d*_*z*_ = 0.26, CI 95% = [− 0.03, 0.56], η_p_^2^ = 0.06, and the interference effect (laboratory: *M* = 25.93 ms, *SD* = 25.13; online: *M* = 28.38 ms, *SD* = 26.08), *t*(180) = 0.65, *p* = 0.520, *d*_*z*_ = 0.10, CI 95% = [− 0.20, 0.39], η_p_^2^ = 0.01 (see Fig. 6).

#### Exploratory Bayes factors for latencies

In exploratory analyses, we wanted to further test the null hypothesis that there is no meaningful difference between the automatic imitation effects of the laboratory and the online sample by applying Bayesian statistics. That is, we report the BF_10_, which is a quantitative measure of evidence in favor of one hypothesis relative to another (i.e., the smaller the BF, the more it argues in favor of the null hypothesis; see Schönbrodt & Wagenmakers, 2018). We calculated the BF_10_ with the default priors (Cauchy prior width* r* = 0.707) in JASP (Version 0.11.1.0; JASP Team, 2019). The BF_10_ values ranged from anecdotal evidence for the null hypothesis to substantial evidence for the null hypothesis, with a BF_10_ = 0.45 for the difference between the congruency effects, a BF_10_ = 0.70 for the difference between the facilitation effects, and a BF_10_ = 0.20 for the difference between the interference effects (cf. Jeffreys, 1998). Eventually, the results of the Bayesian analyses are somewhat inconclusive, mainly because our sample was not sufficiently large for this kind of analysis (Rouder et al., 2009; Schönbrodt & Wagenmakers, 2018).

#### Error rates 

First, we conducted the preregistered tests.^2^ The results of these tests mirrored those of the latencies. In the online sample we found the congruency effect: The participants committed fewer errors in congruent trials (*M* = 0.63%, *SD* = 0.85) than in incongruent trials (*M* = 3.17%, *SD* = 2.60), *t*(83) = 9.61, *p* < 0.001, *d*_*z*_ = 1.05, CI 95% = [0.78, 1.31], η_p_^2^ = 0.53. Moreover, we found a facilitation effect: The participants made fewer errors in congruent trials (*M* = 0.63%, *SD* = 0.85) than in neutral trials (*M* = 1.02%, *SD* = 1.19), *t*(83) = 3.86, *p* < 0.001, *d*_*z*_ = 0.42, CI 95% = [0.20, 0.64], η_p_^2^ = 0.15. Finally, we found an interference effect, as participants made fewer errors in neutral trials (*M* = 1.02%, *SD* = 1.19) than in incongruent trials (*M* = 3.17%, *SD* = 2.60), *t*(83) = 8.77, *p* < 0.001, *d*_*z*_ = 0.96, CI 95% = [0.70, 1.21], η_p_^2^ = 0.48.

In the laboratory sample we found all three imitation effects for the error rates as well. The congruency effect: The participants made fewer errors in congruent trials (*M* = 0.85%, *SD* = 1.06) than in incongruent trials (*M* = 2.90%, *SD* = 2.16), *t*(97) = 9.48, *p* < 0.001, *d*_*z*_ = 0.96, CI 95% = [0.72, 1.20], η_p_^2^ = 0.48. The facilitation effect: The participants committed fewer errors in congruent trials (*M* = 0.85%, *SD* = 1.06) than in neutral trials (*M* = 1.17%, *SD* = 1.06), *t*(97) = 3.29, *p* < 0.001, *d*_*z*_ = 0.33, CI 95% = [0.13, 0.53], η_p_^2^ = 0.10. And the interference effect: The participants made fewer errors in neutral (*M* = 1.17%, *SD* = 1.06) than in incongruent trials (*M* = 2.90%, *SD* = 2.16), *t*(97) = 8.46, *p* < 0.001, *d*_*z*_ = 0.86, CI 95% = [0.62, 1.08], η_p_^2^ = 0.43.

**Fig. 7:**
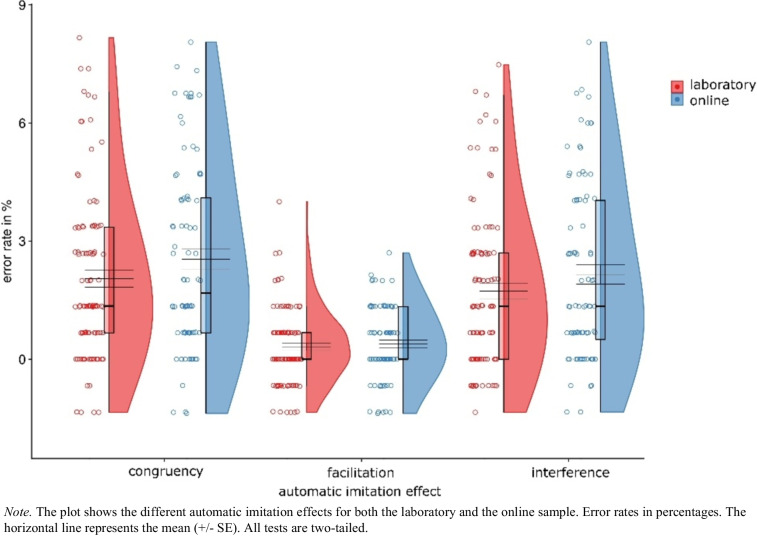
Error rate comparison between online and laboratory samples

A direct comparison of the typical imitation-inhibition effects revealed no difference in the error rates between the laboratory and online samples in terms of the congruency effect (laboratory: *M* = 2.05%, *SD* = 2.14; online: *M* = 2.54%, *SD* = 2.42), *t*(180) = 1.46, *p* = 0.145, *d*_*z*_ = 0.22, CI 95% = [− 0.08, 0.51], η_p_^2^ = 0.05, the facilitation effect (laboratory: *M* = 0.32%, *SD* = 0.95; online: *M* = 0.39%, *SD* = 0.92), *t*(180) = 0.50, *p* = 0.618, *d*_*z*_ = 0.07, CI 95% = [− 0.22, 0.37], η_p_^2^ = 0.005, or the interference effect (laboratory: *M* = 1.73%, *SD* = 2.03; online: *M* = 2.16%, *SD* = 2.25), *t*(180) = 1.34, *p* = 0.182, *d*_*z*_ = 0.20, CI 95% = [− 0.09, 0.49], η_p_^2^ = 0.04 (see Fig. 7).

#### Exploratory Bayes factors for error rates

In another exploratory analysis, we also tested the null hypothesis that there is no meaningful difference between the automatic imitation effects of the laboratory and the online sample with the error rates by applying Bayesian statistics. We again calculated the BF_10_ with the default priors (Cauchy prior width *r* = 0.707) in JASP (Version 0.11.1.0; JASP Team, 2019). The BF_10_ values ranged from anecdotal evidence for the null hypothesis to substantial evidence for the null hypothesis, with a BF_10_ = 0.44 for the difference between the congruency effects, a BF_10_ = 0.18 for the difference between the facilitation effects, and a BF_10_ = 0.37 for the difference between the interference effects (cf. Jeffreys, 1998). As for the latencies, the results of the Bayesian analyses are somewhat inconclusive for the error rates as well, which is mainly because our sample was not sufficiently large for this kind of analysis (Rouder et al., 2009; Schönbrodt & Wagenmakers, 2018).

#### **Reliabilities**

As can be seen in Table 1, when descriptively comparing the reliability, the Spearman–Brown coefficients ρ* of the online version of the imitation-inhibition task are very similar to those of the laboratory-based task—often almost identical. The negative reliabilities of the facilitation effect in the error rates can be best explained by the overall low number of errors, which possibly led to a low correlation between the split-half trials (Krus & Helmstadter, 1993).

**Table 1:** Reliabilities of the online and laboratory samples

	Latencies		Error rates	
	Laboratory	Online	Laboratory	Online
Congruency	ρ* = 0.81	ρ* = 0.81	ρ* = 0.62	ρ* = 0.60
Facilitation	ρ* = 0.41	ρ* = 0.38	ρ* = − 0.03	ρ* = − 0.51
Interference	ρ* = 0.61	ρ* = 0.65	ρ* = 0.54	ρ* = 0.48

ρ* is the Spearman–Brown coefficient. The split-half reliabilities are based on odd and even trials.

#### Explorative analyses

Similarly, as in Experiment 1, we conducted different additional exploratory analyses. These analyses again showed no influence of the ratio between keyboard and screen, the keyboard participants had used, the browser participants had used, handedness, whether they had used an external monitor or a laptop, or the number of repetitions in the first and the second practice block on the imitation-inhibition indices in terms of latencies, *F*s < 2.07, *p*s > 0.072, or error rates, *F*s < 2.08, *p*s > 0.070.

### Discussion

As in Experiment 1, Experiment 2 found the typical imitation-inhibition effects with the online version of the imitation-inhibition task. When comparing the effects with a laboratory sample, there were no differences in terms of effect size and reliability. Even though we did not have a hypothesis and did not perform a direct analysis on this matter, it is still interesting to note that the reaction times in the online sample are descriptively slower than in the laboratory sample. Similarly, the error rate is descriptively larger online than in the laboratory sample. In line with the results obtained in Experiment 1, the results were furthermore not influenced by the ratio between participants’ keyboard and their screen, the keyboard they had used, their handedness, whether they used an external monitor or a laptop, or the number of repetitions in one of the practice blocks. This again illustrates the robustness of the task.

In sum, Experiments 1 and 2 demonstrate that the online version of the imitation-inhibition task produces effects that are comparable to those of laboratory experiments. Yet an open question is whether the online version is sensitive to detect crucial moderator influences. To shed light on this open question, we conducted Experiments 3 and 4.

## Experiment 3

A disadvantage of our previous experiments is that the stimuli confounded imitative with spatial compatibility. That is, participants’ finger movements were (in)congruent not only with the model’s effector (i.e., index and middle finger), but also with the spatial location of the finger movement (i.e., left and right). The goal of Experiment 3 was to test whether the online task produces reliable effects even when controlling for spatial compatibility.

Previous research conducted in the laboratory found that effects of imitative compatibility still occur when spatial compatibility is controlled (e.g., Catmur & Heyes, 2011; Cracco et al., 2018a, 2018b; Jiménez et al., 2012). To test whether the same is true for the online task, we applied a procedure in line with previous research (Bertenthal et al., 2006; Boyer et al., 2012; Catmur & Heyes, 2011). That is, we presented participants not only with left hands but also with right hands (i.e., mirrored left hands) in Experiment 3. This way, we can separately compute imitative compatibility and spatial compatibility effects.

## Method

### Participants 

In order to detect even small effects with a high power, we performed an a priori power calculation: For an effect size of η^2^ = 0.025 with a power of β = 0.90, we needed 72 participants. To compensate for potential dropouts, we preregistered a sample of 100 participants. We recruited the 100 participants via Amazon’s Mechanical Turk in return for compensation of $1.00. The Hit Approval Rate and the location were the same as in Experiment 1.

In line with our preregistration (https://aspredicted.org/tgc6-39ds.pdf), data from a total of seven participants were excluded from data analysis because they had less than 33 valid trials in one or more of the conditions (*n* = 4), or did not use the right hand during the experimental phase (*n* = 4). One participant met both criteria. The final online sample consisted of 93 participants (41 female, 46 male, 6 other) with age ranging from 21 to 75 years (*M* = 38.58, *SD* = 11.66). Ten participants were left-handed and 83 participants were right-handed.

**Fig. 8:**
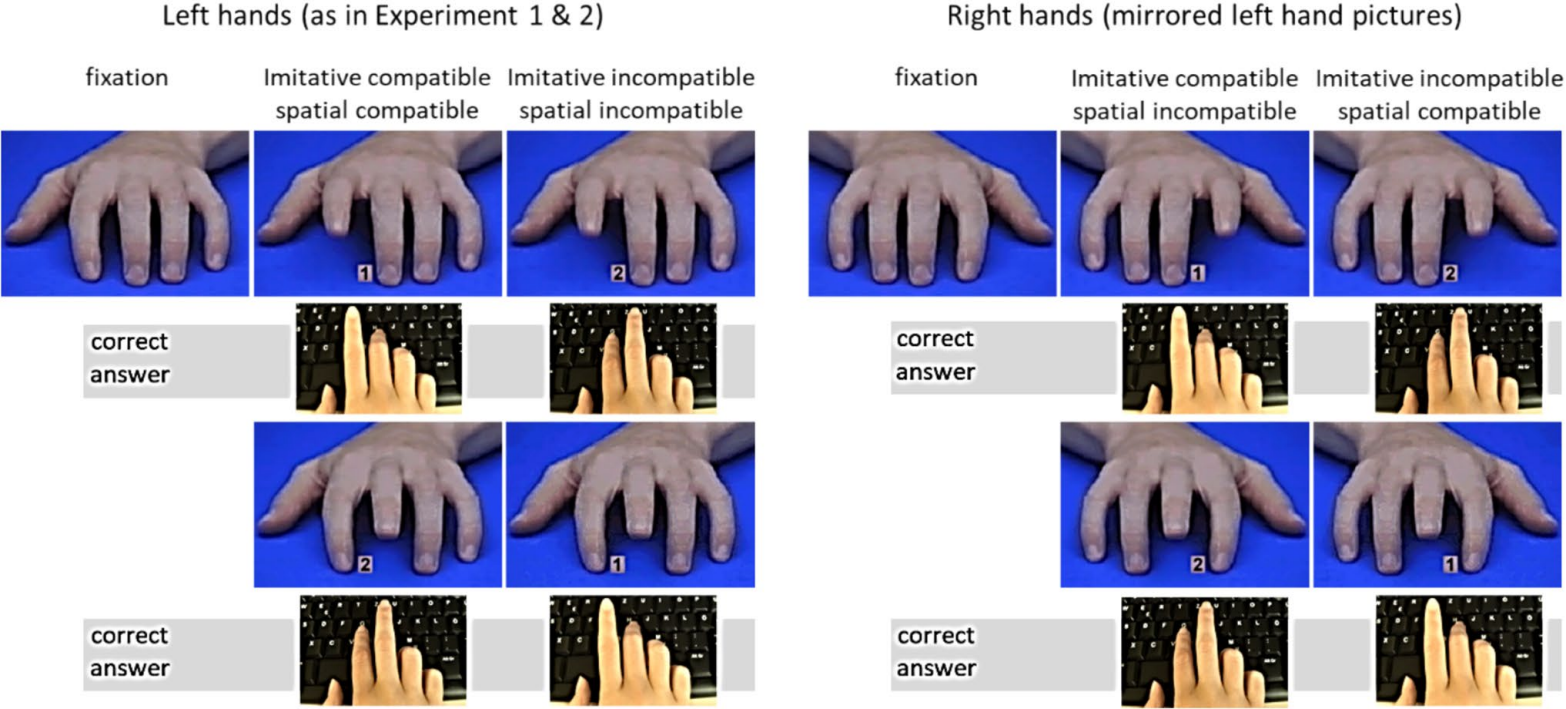
Illustration of the pictures used in their normal and mirrored form

**Procedure.** The procedure in the online task was the same as in Experiments 1 and 2, except that both left and right hands were presented on the screen in the second practice block and also in the experimental block (see Fig. 8). In addition, the task comprised only congruent and incongruent but no neutral trials.

The first practice block remained exactly the same as in Experiments 1 and 2. The second practice block consisted of 16 trials in total (8 left hands and 8 right hands), and participants had to repeat it until they committed fewer than eight errors. The experimental blocks consisted of 200 trials, with 50 trials for each of the four conditions. The trials were presented in randomized order across five blocks (40 trials each). Participants could take a self-paced break between each block. To prepare the data for analysis, we preregistered the same trial-based exclusion criteria as in the other experiments. That is, we discarded trials with reaction times below 100 ms (0.77%). We did not remove latencies below and above 3 *SD*s of the participant’s mean, as there were no such trials detected. For the analyses of the latencies, we removed erroneous trials as well (9.02%).

### Results

#### Latencies

**Fig. 9:**
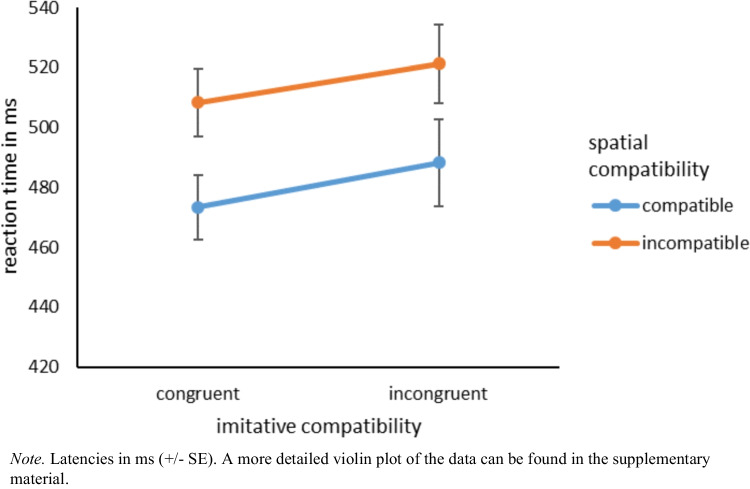
Reaction times imitative compatibility × spatial compatibility

To test our hypotheses, we firstly conducted a 2 (imitative compatibility: congruent vs. incongruent) × 2 (spatial compatibility: compatible vs. incompatible) repeated-measures ANOVA for the latencies. We found a main effect for imitative compatibility, *F*(1, 92) = 12.39, *p* < 0.001, η_p_^2^ = 0.12, indicating that participants responded faster in imitative congruent trials (*M* = 490.89 ms, *SD* = 106.23) than in imitative incongruent trials (*M* = 504.72 ms, *SD* = 132.49). Also, the main effect for spatial compatibility was significant, *F*(1, 92) = 229.28, *p* < 0.001, η_p_^2^ = 0.71. This means that participants responded faster in spatially compatible trials (*M* = 480.82 ms, *SD* = 120.28) than in spatially incompatible trials (*M* = 514.79 ms, *SD* = 117.86). The interaction was not significant, *F*(1, 92) = 0.20, *p* = 0.656, η_p_^2^ = 0.002. In line with the nonsignificant interaction, the preregistered planned contrast analyses showed that irrespective of spatial compatibility, participants responded significantly faster to imitative congruent movements than to imitative incongruent movements in both the spatially compatible condition, *F*(1, 92) = 7.78, *p* = 0.006, η_p_^2^ = 0.08, and the spatially incompatible condition, *F*(1, 92) = 13.61, *p* < 0.001, η_p_^2^ = 0.13, indicating that automatic imitation measured with the online version of the imitation-inhibition task measured imitative behavior even when spatially incompatible movements were presented (see Fig. 9).

#### Error rates

**Fig. 10:**
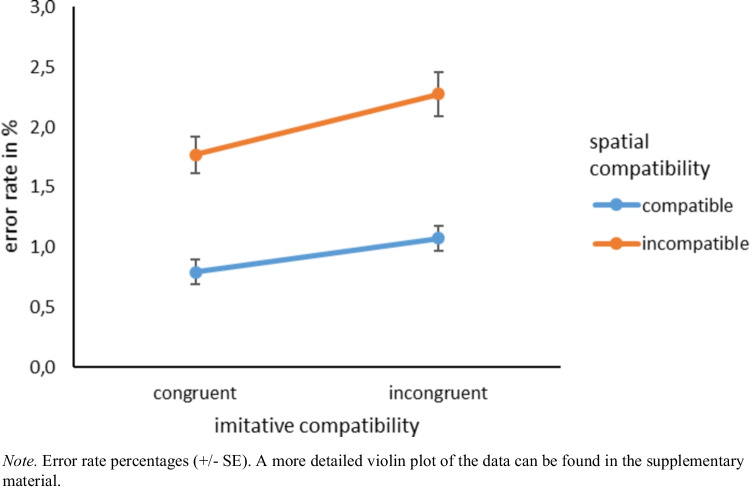
Error rates imitative compatibility × spatial compatibility

The same analyses of the error rates yielded similar effects.^3^ Again, we found both the main effect for imitative compatibility, *F*(1, 92) = 14.20, *p* < 0.001, η_p_^2^ = 0.13, and the main effect for spatial compatibility, *F*(1, 92) = 71.92, *p* < 0.001, η_p_^2^ = 0.44. This means that participants made fewer errors in imitative congruent trials (*M* = 1.28%, *SD* = 1.06) than in imitative incongruent trials (*M* = 1.67%, *SD* = 1.22). Likewise, they made fewer errors in spatially compatible trials (*M* = 0.93%, *SD* = 0.86) than in spatially incompatible trials (*M* = 2.02%, *SD* = 1.47). The interaction was not significant, *F*(1, 92) = 1.92, *p* = 0.169, η_p_^2^ = 0.02. The planned contrasts revealed that irrespective of spatial compatibility, participants made fewer errors in congruent trials than in incongruent trials in both the spatially congruent condition, *F*(1, 92) = 7.04, *p* = 0.009, η_p_^2^ = 0.07, and the spatially incongruent condition, *F*(1, 92) = 10.63, *p* = 0.002, η_p_^2^ = 0.10 (see Fig. 10).

### Discussion

Experiment 3 replicated the typical effects found in the laboratory with the online imitation-inhibition task. That is, in line with previous research conducted in laboratory settings (e.g., Catmur & Heyes, 2011; Cracco et al., 2018a, 2018b; Jiménez et al., 2012), our experiment demonstrated that automatic imitation assessed with the online task is still robust when imitative compatibility is assessed orthogonally to spatial compatibility. In other words, the results of Experiment 3 show that the online version of the imitation-inhibition task is a measure of automatic imitation, because spatial compatibility cannot explain the detected congruency effects. Yet an open question is whether automatic imitation measured with the online version of the imitation-inhibition task as a social process is also modulated by social factors. To investigate this question, we conducted Experiment 4.

## Experiment 4

The purpose of Experiment 4 was to test whether the online imitation-inhibition task can be socially modulated when applying a manipulation that has been frequently used in the laboratory. Specifically, we investigated whether automatic imitation is different for robotic as compared to human hands. In laboratory settings, typically, a smaller automatic imitation effect is detected for robotic hands compared to human hands (Bird et al., 2007; Chaminade & Cheng, 2009; Kilner et al., 2003; 2003; Press et al., 2005, 2006).

### Participants 

To be able to detect even very small effects, we performed an a priori power calculation: For an effect size of η^2^ = 0.01 with a power of β = 0.90, we needed 178 participants. Considering potential dropouts, we recruited 200 participants via Amazon’s Mechanical Turk in return for compensation of $1.00. As for the first three experiments, only MTurkers located in the United States with a Hit Approval Rate over 85% were invited to participate in the study, and participants could only take part in the study with a laptop or computer. We applied the same exclusion criteria as in the first three experiments. In total, data from 96 participants were excluded from data analysis because they did not use the right hand during the experimental phase (*n* = 18) or had less than 33 valid trials in one or more of the conditions (*n* = 88). Thirteen participants met both criteria. Three participants had technical problems, which is why their data was submitted empty.^4^ The final online sample consisted of 104 participants (36 female, 64 male, 3 other, 1 missing) with age ranging from 22 to 69 years (*M* = 38.40, *SD* = 11.42). Six participants were left-handed and 98 participants were right-handed.

### Procedure

**Fig. 11:**
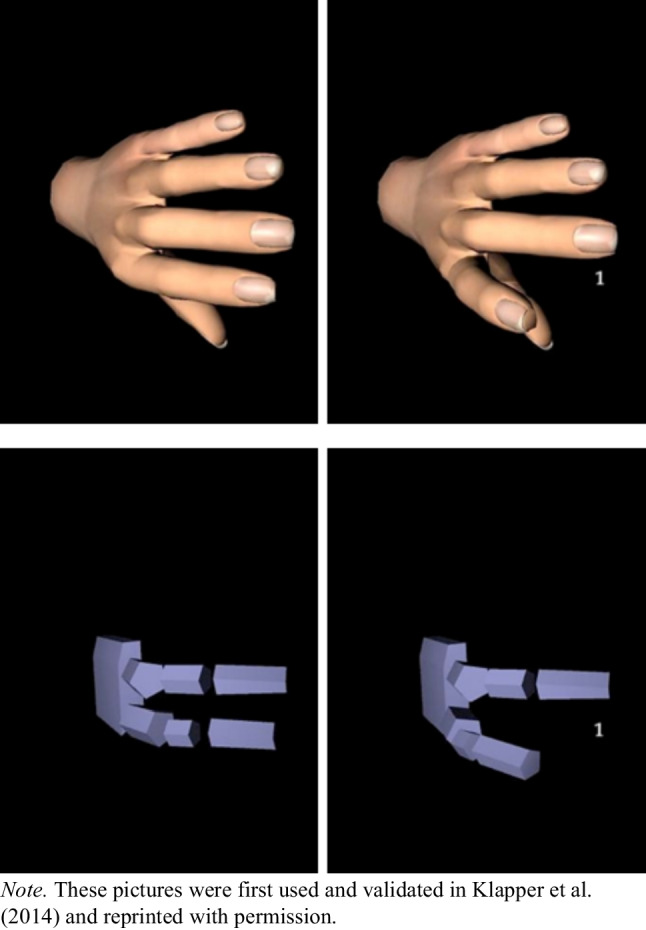
Example pictures of robotic and human hand stimuli used in Experiment 4

The procedure in the online task was similar to the procedure in Experiments 1 and 2 with a few exceptions. That is, we included congruent and incongruent but no neutral trials. The two practice blocks remained the same as in Experiments 1 and 2. Another difference concerned the stimuli presented. Instead of presenting human hands only, in Experiment 4 we presented participants with a robotic and an artificial human hand. We randomized on a trial-by-trial basis whether the human hand or the robotic hand was presented. The hand pictures have already been used in different prior experiments with the imitation-inhibition task (e.g., Klapper et al., 2014). We presented the images rotated by 90°—an often-used technique to reduce the impact of spatial compatibility (for more information, see Cracco et al., 2018a, 2018b).^5^ Example pictures can be found in Fig. 11.

The experimental blocks consisted of 160 trials (40 congruent robotic hand trials, 40 incongruent robotic hand trials, 40 congruent human hand trials, and 40 incongruent human hand trials). Participants could take a self-paced break between each block. To prepare the data for analysis, we used the same trial-based exclusion criteria as in the other three experiments. That is, we removed latencies below (0.14%) and above (1.17%) 3 *SD*s of the participant’s mean. We also discarded trials with reaction times below 100 ms (2.74%). For the analyses of the latencies, we removed erroneous trials as well (18.24%).

### Results

#### Latencies 

**Fig. 12:**
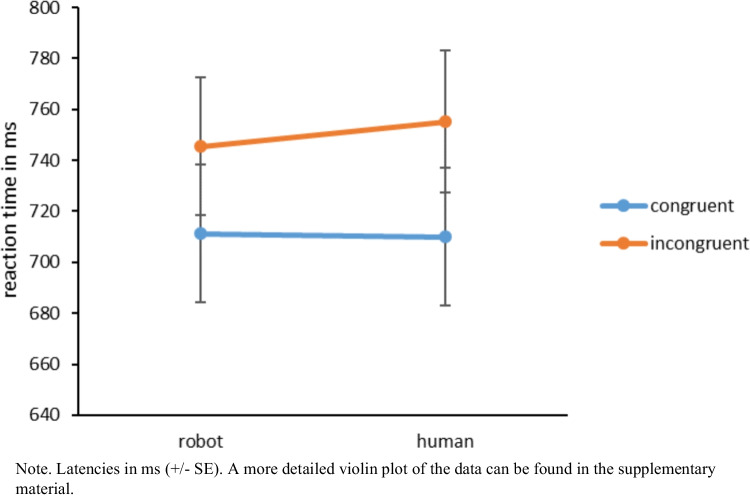
The difference in the trial conditions for the latencies

To test the hypothesis that the automatic imitation effect is stronger for human hands than for robotic hands, we conducted a 2 (imitative compatibility: congruent vs. incongruent) × 2 (hand condition: human vs. robotic) repeated-measures ANOVA for the latencies. We found a main effect for imitative compatibility (i.e., congruency effect), *F*(1, 103) = 146.08, *p* < 0.001, η_p_^2^ = 0.59, indicating that participants responded faster in congruent trials (*M* = 710.63 ms, *SD* = 275.30) than in incongruent trials (*M* = 750.32 ms, *SD* = 280.13). Likewise, the main effect for hand condition was significant, *F*(1, 103) = 7.42, *p* = 0.008, η_p_^2^ = 0.07, which means that participants responded faster in robotic hand trials (*M* = 728.35 ms, *SD* = 275.80) than in human hand trials (*M* = 732.60 ms, *SD* = 323.55). More importantly, the interaction was significant as well: This means that the congruency effect was stronger for human hands (*M* = 45.14, *SD* = 37.32) than robotic hands (*M* = 34.26, *SD* = 40.42), *F*(1, 103) = 7.87, *p* = 0.006, η_p_^2^ = 0.07 (see Fig. 12).

#### Error rates 

We ran the same analyses for the error rates as well.^6^ We again found the main effect for congruency, *F*(1, 103) = 48.19, *p* < 0.001, η_p_^2^ = 0.32, meaning that participants made fewer errors in congruent trials (*M* = 1.13%, *SD* = 1.15) than in incongruent trials (*M* = 2.34%, *SD* = 1.66). We did not find a main effect for the hand condition, *F*(1, 103) = 0.87, *p* = 0.353, η_p_^2^ = 0.008. The error rate between robotic hand trials (*M* = 1.68%, *SD* = 1.21) and human hand trials (*M* = 1.79%, *SD* = 1.30) did not differ significantly this time. The interaction between the congruency and the hand condition was also not significant, *F*(1, 103) = 1.88, *p* = 0.173, η_p_^2^ = 0.02, which means that the congruency effect did not vary between human (*M* = 1.03, *SD* = 2.15) and robotic hands (*M* = 1.38, *SD* = 2.25) within the error rates.

## Discussion

Using the online version of the imitation-inhibition task, Experiment 4 aimed at replicating a social modulation of automatic imitation, which has often been found in laboratory experiments. Similar to typical effects found in the laboratory (e.g., Cracco et al., 2018a, 2018b), individuals imitated human hands more strongly than robotic hands when analyzing the latencies. It is important to note that the reaction times in Experiment 4 are slower than the reaction times detected in our previous experiments. Reasonable explanations for this observation are the mental processing of the stimuli as well as the testing environment. In Experiment 4 we used rather artificial hand pictures as stimuli. These stimuli were the same as Klapper et al. (2014) used. Interestingly, as compared to other experiments using the imitation-inhibition task (for a meta-analysis, see Cracco et al., 2018a, 2018b), Klapper et al. (2014) also found rather slow reaction times. Thus, it might well be that the artificial character of the depicted hands needs more mental resources to process the stimuli, which results in slower reaction times. Another interesting observation is that although Klapper et al. (2014) detected slower reaction times than usually found in the automatic imitation literature, their reaction times were still somewhat faster than those we detected in Experiment 4. As Klapper et al. (2014) assessed reaction times in the laboratory, whereas we measured automatic imitation online, the testing environment might explain why our participants in Experiment 4 responded slower than those assessed by Klapper et al. (2014). Support for this explanation comes from Experiment 2, which found that the reaction times in the online sample were indeed slower than those in the laboratory sample. Taken together, the slower reaction times detected in Experiment 4 might be due to two factors: the mental processing of artificial hands and the online setting of the experiment.

For the error rates, we found the typical congruency effect, but no significant influence of the robotic versus human hand. In this respect, it is important to note that the error rate is often not sensitive to the modulation of imitative behavior (Cracco et al., 2018a, 2018b; Genschow et al., 2017). In addition, due to the exclusions, we did not reach the number of participants that we planned, which might have also contributed to not finding a possible small effect. Thus, one can conclude that the online version of the imitation-inhibition task mirrors the results of typical laboratory experiments investigating the modulation of automatic imitation.

## General discussion

People automatically imitate others’ behaviors (Cracco et al., 2018a, 2018b; Heyes, 2011). The most commonly used task to measure automatic imitation tendencies is the imitation-inhibition task, a task typically used in costly and time-consuming laboratory settings. In this article we present an online version of the imitation-inhibition task, which can be implemented using online survey software (e.g., Qualtrics). In four experiments, we validated the online version of the imitation-inhibition task. The results show that the task works efficiently (Experiment 1) and achieves similar results in terms of effect size and reliability as compared to laboratory settings (Experiment 2). Moreover, the online version of the imitation-inhibition task taps similarly into social processes as its laboratory equivalent, since it reliably detects automatic imitation even when controlling for spatial compatibility (Experiment 3), and is modulated by animacy in the sense that individuals imitate robotic finger movements less strongly than human finger movements (Experiment 4).

To implement the online version of the imitation-inhibition task, we provide two solutions. First, on the OSF, we provide programmed versions that run by themselves on any server. Second, we provide a solution that allows the imitation-inhibition task to be run in the Qualtrics online survey tool. Although common survey software is not intentionally designed to perform reaction time tasks such as the imitation-inhibition task, we have shown that it can be used very easily when implementing the additional codes. On OSF, we provide all the codes necessary to run the task in Qualtrics and on a server along with detailed tutorials and templates. Additionally, we present analysis scripts to preprocess and analyze the data in R.

### Practical advantages of the online version of the imitation-inhibition task

The advantages of the online version of the imitation-inhibition task are manifold. First, the imitation-inhibition task with the survey software solution we provide here can be very easily and conveniently implemented and adjusted by researchers with limited programming knowledge. Therefore, the task can also be used for teaching purposes and in student research projects.

Second, when using traditional programming software solutions that allow reaction time-based experiments to be run online, participants sometimes need to install or download a file or software, or redirection to external platforms might be necessary. As many participants are hesitant to download such files, the range of participants that take part in these kinds of experiments is limited. This does not apply to the programmed solutions we provide here, as participants do not need to download any file, nor are they redirected to external platforms, ensuring that no data will be lost.

Third, the task works equally well for all computers (either laptops or stationary PCs), keyboards, laptop vs. external monitors, and browsers, as we found no difference in automatic imitation with respect to the participants’ equipment in either the first or the second experiment.

### Broad scope of the online version of the imitation-inhibition task

Besides the practical advantages, the online imitation-inhibition task offers the potential to increase the use of the imitation-inhibition task in several ways, thereby broadening the scope of research questions. First, as noted earlier, imitation studies are typically conducted with small, in-person samples in the laboratory (e.g., Liepelt & Brass, 2010). However, such small samples are less informative, lead to inaccurate parameter estimates, and have been criticized as a factor leading to replicability issues (Brandt et al., 2014; Szucs & Ioannidis, 2017). The online imitation-inhibition task offers the possibility to conduct high-powered experiments with little effort. This may be especially useful for research questions for which small effects are expected and thus large samples are needed. For example, the online imitation-inhibition task can help resolve the debate concerning the degree to which social factors affect automatic imitation. Although there are a several studies showing the influence of social variables on automatic imitation (e.g., Bird et al., 2007; Cracco et al., 2015; Hogeveen & Obhi, 2013; Leighton et al., 2010; Liepelt & Brass, 2010; Rauchbauer et al., 2016), other studies have had trouble replicating these findings (e.g., Butler et al., 2015; Galang & Obhi, 2020; Genschow et al., 2017; Müller et al., 2013; Newey et al., 2019). A reasonable assumption for the mixed results is that the expected effect size for these findings is most likely small. As the online imitation-inhibition task allows the testing of large samples in an effortless manner, it is well suited for investigating research questions for which a small effect size is expected, thereby shedding light on some unresolved debates.

Second, and related to the first point, the imitation-inhibition task presented here is well suited for conducting high-powered replications of previous experiments. Replications sometimes require more participants than were actually included in the original articles, if the original study was not sufficiently powered (Brandt et al., 2014; Simonsohn, 2015). The collection of such large samples is made possible and simplified by the online version of the task.

Third, the online version of the imitation-inhibition task offers new avenues for a diverse set of research questions that cannot be answered in the laboratory. For example, the question of which cultures imitate more or less can only be tested by measuring imitation in many different countries. Such an approach is difficult to implement in laboratory settings, but rather easily achieved when applying the online task.

Fourth, the basic structure of the online version of the imitation-inhibition task we presented here can, in principle, be used for any response-compatibility task, such as Stroop tasks (Stroop, 1935), Simon tasks (Simon & Rudell, 1967), or Flanker tasks (Eriksen & Eriksen, 1974). As all these tasks involve a similar structure and procedure as the imitation-inhibition task, researchers could use the online version of the imitation-inhibition task by exchanging the stimuli, adapting the instructions, and adjusting the number of trials (if needed). When adapting the online version of the imitation-inhibition task to assess other response-compatibility tasks, we advise researchers to use similar exercise blocks as we did to familiarize participants with the task and to guarantee that all participants understand the instructions before they start with the first experimental block.

Fifth, our findings contribute to the debate regarding whether online studies lead to similarly strong and reliable effects as laboratory experiments (e.g., Douglas et al., 2023; Germine et al., 2012; MacInnis et al., 2018; Plant, 2016; van Steenbergen & Bocanegra, 2016). Our findings indicate that by using the method we validated here, automatic imitation effects assessed in online settings are comparable in terms of effect size and reliability with the effects detected in laboratory settings. We believe that similar results can be expected when applying the logic of our task to other response-compatibility tasks. This would indicate that online studies are well suited for response-compatibility tasks, allowing researchers to test large samples within a short amount of time with limited costs.

Sixth, psychological research has been frequently criticized in recent times for collecting WEIRD samples (i.e., participants from western, educated, industrialized, rich, and democratic nations), especially the United States (Cheon et al., 2020; Muthukrishna et al., 2020; Rad et al., 2018). Thus, psychological data do not really represent the entire world population (Muthukrishna et al., 2020). With an online version of the imitation-inhibition task, it now becomes easier to collect representative samples from different parts of the world via panel providers, thus avoiding the use of typical college student samples.

### Limitations

Despite its advantages, the online imitation-inhibition task may have some limitations that we would like to mention here. First of all, it should be noted that there may also be situations in which an in-person imitation-inhibition task in the laboratory is desirable. For example, researchers may wish to involve other observed behaviors that cannot be measured online or may desire greater control over the environment (e.g., the location of the experiment, correct handling of the experimental material, possibility to ask questions in case of ambiguity, more complicated manipulations). Nevertheless, researchers who wish to perform the imitation-inhibition task in the laboratory could theoretically use the survey software online imitation-inhibition task or the server-based version in the laboratory as well.

Second, and related to the first point, is that the online task can only be used to measure finger lifting movements. Previous research sometimes measured imitation of other movements, such as hand opening and closing motions (e.g., Press et al., 2006) or finger lowering instead of finger lifting (e.g., Boyer et al., 2012). Given that our task can only detect key releases made on a computer keyboard, the range of movements is limited to actions that involve key releases (or with adjustment of the JavaScript also key presses). Any other movement behavior should be measured in the laboratory or with a different task. However, as most researchers use finger lifting movements to study automatic imitation, our task is nevertheless adaptable for most research questions on automatic imitation.

Third, from the comparison of the samples of Experiment 2 and especially the sample of Experiment 4, we can derive one primary caution: Although the imitation-inhibition task itself produces good and comparable results for both students in the lab sample and crowdsource participants (e.g., MTurk) online, a somewhat larger proportion of MTurk participants took the imitation-inhibition task less seriously. That is, with the same exclusion criteria, the exclusion rate was considerably higher for the online sample than for the laboratory (i.e., highest for the fourth experiment), as some participants made more intentional errors online (e.g., not raising their finger at all), which is why they did not achieve comparable trial numbers in the different trial conditions. As lower-quality data compared to laboratory research is a known problem in online research (De Man et al., 2021; Gosling & Mason, 2015; Gosling et al., 2004), we advise researchers to preregister the exclusion criteria in experiments with the online imitation-inhibition task and to take into account a likely higher exclusion rate when calculating the sample, as is common practice in other online experiments. At least when applying the exclusion criteria we used here, the results detected with the imitation-inhibition task are similar to those of its laboratory equivalent. In addition, it might also make sense to think about online panel providers other than MTurk, since MTurk is known to collect lower-quality data compared to other providers (e.g., Chmielewski & Kucker, 2020; Woo et al., 2015). Furthermore, we recommend implementing the same strict practice phases we used in our experiments (i.e., participants cannot start with the actual experimental phase if they have not successfully completed the minimum number of trials in both practice phases) to ensure that even participants who did not read the instructions carefully will understand the task.

Fifth, participants can only take part on computers with keyboards; participants who prefer tablets or mobile phones cannot participate in the online version of the imitation-inhibition task. Nevertheless, one does not have to consider this as a limitation, since this is a key feature of the task, and it might not even be considered automatic imitation when a tablet or a mobile phone is used to conduct an imitation-inhibition task, either in the laboratory or online.

A final limitation is that the codes we provided currently work without adaptation only when running experiments on Qualtrics or a personal server. Nevertheless, based on the codes and tools we provide on OSF, we are confident that an implementation within other online survey platforms or freely available server providers (e.g., MindProbe, https://mindprobe.eu/ or Cognition.run, https://www.cognition.run/) is feasible with only slight adjustments.

### Conclusion

The validation of the online imitation-inhibition task allows researchers across the globe to investigate imitative behavior online without much effort. This moves research away from the same samples collected on campus and allows for testing a wide range of research questions in a variety of different samples and cultures. The online imitation-inhibition task is a reliable and valid method for conducting imitation research that reduces costs, enables recruitment of large online samples, and simplifies data management. Compared to laboratory samples, the effects obtained with online measurements are similar in terms of size and reliability. Thus, the online imitation-inhibition task is a well-performing alternative to its laboratory version. With the use of the materials we have provided here, the implementation of the online procedure will be simplified to facilitate high-quality imitation research in the future.

## Supplementary Information

Below is the link to the electronic supplementary material.

Supplementary file1 (DOCX 1.01 MB)

## Data Availability

The datasets generated and analyzed during the current study are available in the OSF repository “Online Imitation-Inhibition Task” [https://osf.io/q7fju/].

## Footnotes

1 To deal with the slightly varying raw percentage error rates, we also performed an arcsine square root transformation and then repeated the error rate analyses, which did not change the results. Further details can be found in the supplementary materials.

2 To deal with the slightly varying raw percentage error rates, we also performed an arcsine square root transformation and then repeated the error rate analyses, which did not change the results. Further details can be found in the supplementary materials.

3 To deal with the slightly varying raw percentage error rates, we also performed an arcsine square root transformation and then repeated the error rate analyses, which did not change the results. Further details can be found in the supplementary materials.

4 Please note that we had to exclude more participants than in our previous experiments, even though we used the same exclusion criteria as in the first three experiments. A potential reason for the higher dropout is that participants were less motivated and attentive as compared to our previous experiments, as there were many participants who did not respond at all to nearly one third of all trials. Also, a couple of these participants had to repeat one or both practice phases more than 10 times, which also suggests that they were neither motivated nor attentive to the study.

5 To obtain further information about the social modulation, we also asked three questions about the perceived similarity of the hands individually shown and randomized before the demographics. Specifically, we again presented the different hands and asked the following: “How similar or dissimilar do you find the hands?” “How similar is this hand to yours?” (for robotic and human hand). Participants rated all questions on sliders from 1 (“very dissimilar”) to 100 (“very similar”). They perceived their own hand to be more similar to the artificial human hand (*M* = 77.00, *SD* = 21.54) than to the robotic hand (*M* = 33.04, *SD* = 35.84), *t*(103) = 12.40, *p* < .001, *d*_*z*_ = 1.22, CI 95% [0.96, 1.47], although they found the hands neither similar nor dissimilar to each other (*M* = 44.37, *SD* = 34.76), *t*(103) = − 1.65, *p* = .101, *d*_*z*_ = − 0.16, CI 95% [− 0.36, 0.03].

6 To deal with the slightly varying raw percentage error rates, we also performed an arcsine square root transformation and then repeated the error rate analyses, which did not change the results. Further details can be found in the supplementary materials.

## Code availability

The codes generated during the current study are available in the OSF repository “Online Imitation-Inhibition Task” [https://osf.io/q7fju/].

## Declarations

### Open practices statement

The data and materials for all four experiments are available at OSF (https://osf.io/q7fju/). Experiment 1 (https://aspredicted.org/k9zz-p62v.pdf), Experiment 2 (https://aspredicted.org/775h-8h4y.pdf), and Experiment 3 (https://aspredicted.org/tgc6-39ds.pdf) were preregistered.

### Author note

Inquiries regarding the plugins should be sent to Emiel Cracco (Emiel.Cracco@UGent.be). Inquiries regarding the validation of the online task should be sent to Mareike Westfal (mareike.westfal@leuphana.de).

Inquiries regarding the whole code and the structure of the online task can be sent to both.
