# Supplementary material for: Validation of an online imitation-inhibition task
Source: Behav Res Methods. 2025 Jan 30;57(2):80. doi: 10.3758/s13428-024-02557-3 (PMC11782408; doi:10.3758/s13428-024-02557-3)
Supplement: Supplementary file 1 — Supplementary file1 (DOCX 1.01 MB) [file 13428_2024_2557_MOESM1_ESM.docx]

Supplementary material for

Validation of an Online Imitation-Inhibition Task

Mareike Westfal

(Leuphana University Lueneburg, Germany)

Emiel Cracco

(Ghent University, Belgium)

Jan Crusius

(University of Greifswald, Germany)

Oliver Genschow

(Leuphana University Lueneburg, Germany)

**This document includes**

Additional analyses

References

**Explorative Analysis of the Correlation Between Age and Reaction Time Automatic Imitation Effects.**

Cracco and colleagues (2018) tested in their meta-analysis whether age correlates with automatic imitation. The results did not reveal any meaningful relationship. This might be, because the meta-analysis could only assess the effect of participants’ mean age of mainly students on imitation. Our sample allowed analyzing in an exploratory fashion whether the wide age range of the participants in a more diverse sample relates to the automatic imitation effects. We analyzed the reaction time correlation between age and the automatic imitation effects once as raw units, that is, as a pure difference between the respective trial conditions (i.e., generated by subtracting the trial conditions from each other) and once as a relative difference between the trial conditions (i.e., generated by dividing the trial conditions by each other).

Because we conducted several studies, we decided to calculate a mini meta-analysis for the correlation of age with the congruency effect (i.e., the effect that directly reflects the strength of automatic imitation). We opted for a meta-analysis with random effects because first, we wanted to enable generalizability of the results to other studies and second, because the four studies addressed different research questions and had correspondingly heterogeneous designs, which could have influenced the effect sizes (Borenstein et al., 2010; Dettori et al., 2022; Field, 2001). For the third study, we averaged the congruency effect across spatially compatible and incompatible conditions. For the fourth study, we only included the congruency effect of the human hand to avoid biasing the results.

We analyzed the data with R (R Core Team, 2022; version 4.3.0) using the metafor package (Viechtbauer, 2010).

**Reaction Times**

The results across Experiments 1–4 indicated that the congruency effect did not correlate with age, neither for the pure difference between the respective trial conditions, *r_pooled_* = 0.09, 95% CI [− 0.02, 0.20], *SE* = 0.06, *z* = 1.67, *p* = .094, nor for the relative difference between the trial conditions, *r_pooled_* = 0.08, 95% CI [− 0.01, 0.17], *SE* = 0.05, *z* = 1.66, *p* = .097. As can be seen in Figure 1 for the pure difference and in Figure 2 for the relative difference, the correlation between age and the congruency effect varied but never exceeded a weak correlation.

**Figure 1**

*Forest plot for the Correlation Between Age and Congruency Effect for Reaction Times (Pure Difference Between Conditions)*


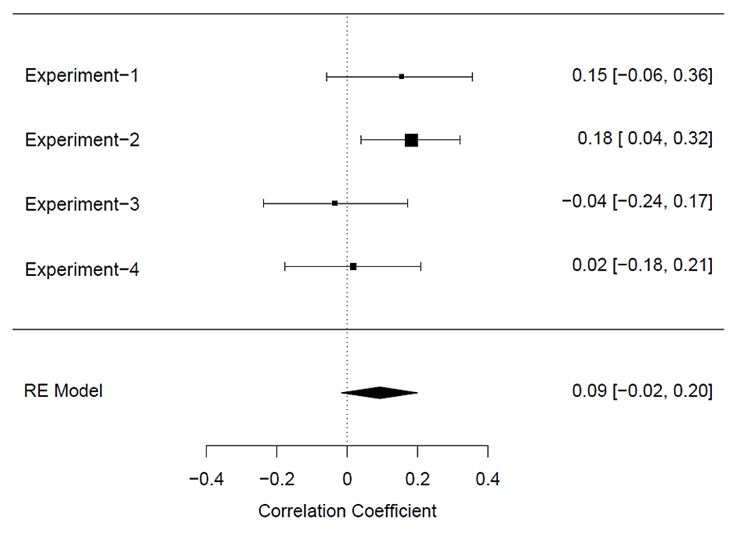


**Figure 2**

*Forest plot for the Correlation Between Age and Congruency Effect for Reaction Times (Relative Difference Between Conditions)*


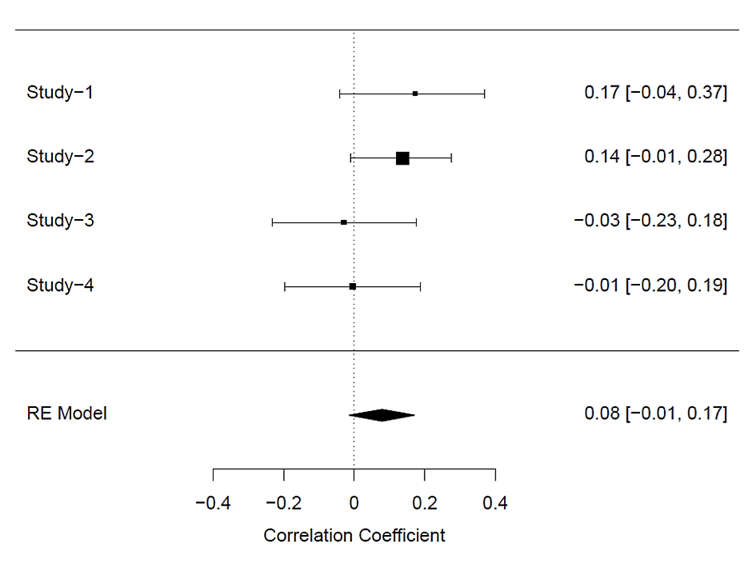


For the facilitation and interference effects, it did not make sense to calculate a mini meta-analysis, as we only have two studies reporting these effects. Therefore, we present the results here in Table 1. For Experiment 2, the correlation of age with the facilitation effect is significant, *r* = .17, *p* = .020. However, since it was not significant in Experiment 1, the correlation would have to be replicated again before a stable effect can be assumed. Otherwise, the correlations are not significant, regardless of whether we correlate relative or pure differences of the respective automatic imitation effects with the age, *r* < 0.17, *p* > .067.

**Table 1**

*Correlations Between Age and Automatic Imitation Effects (Reaction Times)*

|  |  | RT facilitation | RT interference | Relative RT facilitation | Relative RT interference |
| --- | --- | --- | --- | --- | --- |
| Experiment 1 | age | 0,08 | 0,16 | 0,09 | 0,18 |
| Experiment 2 | age | 0,17* | 0,12 | 0,13 | 0,08 |

*Note.* The correlations of the imitation effects are shown as raw units (e.g., RT facilitation = congruent trials – neutral trials) and as relative change (e.g., Relative RT facilitation = congruent trials / neutral trials).

** p* < .05, two-tailed.

**Error Rates**

For the error rates we conducted the same mini meta-analysis. The results indicated that the congruency effect of the error rates did not correlate with age, neither for the pure difference between the respective trial conditions, *r_pooled_* = -0.008, 95% CI [− 0.10, 0.08], *SE* = 0.05, *z* = -0.17, *p* = .865, nor for the relative difference between the trial conditions, *r_pooled_* = 0.05, 95% CI [− 0.07, 0.17], *SE* = 0.06, *z* = 0.85, *p* = .395. As can be seen in Figure 3 and in Figure 4, the correlation between age and the congruency effect of the relative and the pure difference never reached significance.

**Figure 3**

*Forest plot for the Correlation Between Age and Congruency Effect for Error Rates (Pure Difference Between Conditions)*


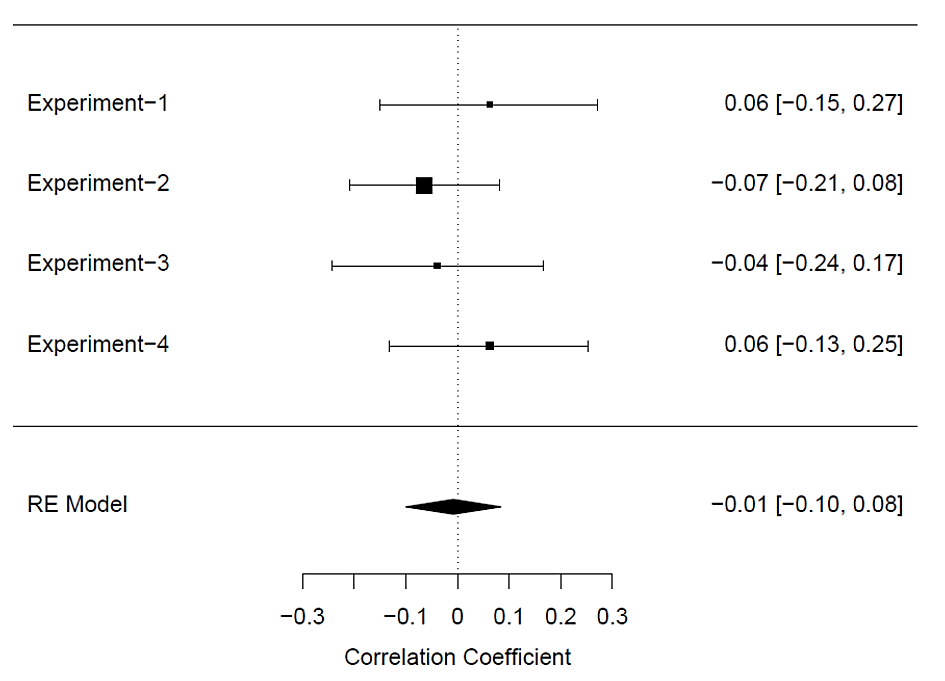


**Figure 4**

*Forest plot for the Correlation Between Age and Congruency Effect for Error Rates (Relative Difference Between Conditions)*


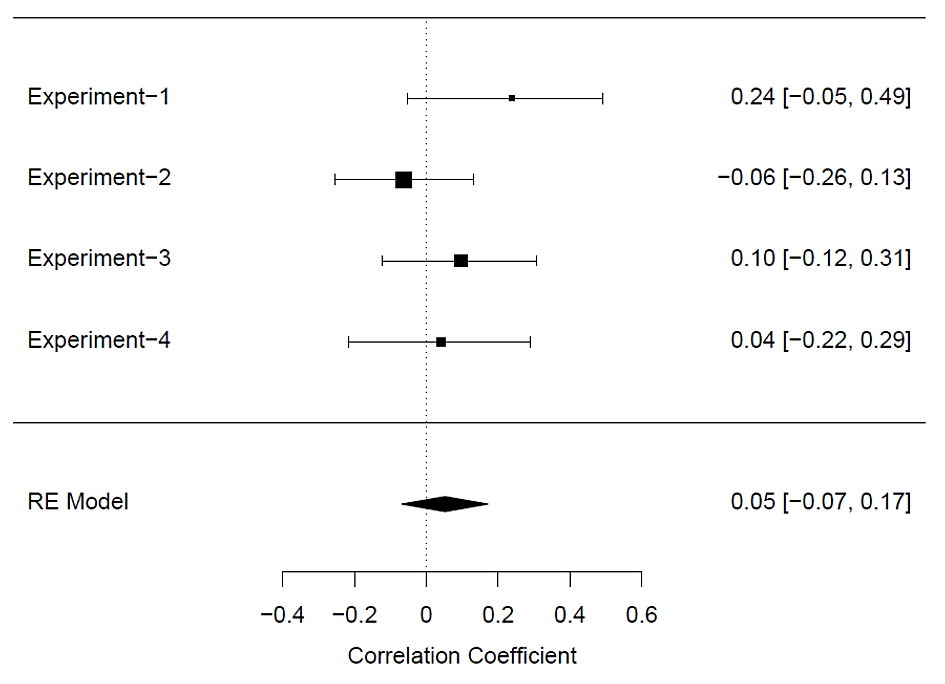


The results of the correlations of the facilitation and interference effect with age are presented in Table 2. The correlations are not significant, regardless of whether we correlate relative or pure differences of conditions for the respective automatic imitation effect with the age, *r* < 0.24, *p* > .108.

**Table 2**

*Correlations Between Age and Automatic Imitation Effects (Error Rates)*

|  |  | RT facilitation | RT interference | Relative RT facilitation | Relative RT interference |
| --- | --- | --- | --- | --- | --- |
| Experiment 1 | age | 0,007 | 0,07 | -0,004 | 0,13 |
| Experiment 2 | age | -0,11 | -0,02 | 0,04 | -0,07 |

*Note.* The correlations of the imitation effects are shown as raw units (e.g., RT facilitation = congruent trials – neutral trials) and as relative change (e.g., Relative RT facilitation = congruent trials / neutral trials).

**Analyses With Adjusted Error Rate Percentages**

Although the reported error rates are comparable with the error rates reported in previous research using the imitation-inhibition task (for a meta-analysis, see Cracco, Bardi, et al., 2018), one may argue that the error rate percentages are rather low and thus unsuited for the analyses we applied. For data based on a binomial distribution (e.g. true, false), the variance is a function of the mean, which can make calculations problematic if the values also vary little (e.g. few to no errors are made). In order to obtain a variance-stabilized mean value, transformations can be performed. We thus reran all the analyses on the error rates (including the explorative analyses) with adjusted error rate percentages. Specifically, we used the arcsine-square-root transformation (Ahrens et al., 1990; Laurencelle & Cousineau, 2023). We applied the transformation to all error rate percentages per participant.

**Experiment 1**

**Error Rates.** We detected a congruency effect as participants made fewer errors in congruent trials (*M* = 0.06 % _asrt_, *SD* = 0.06) than in incongruent trials (*M* = 0.17 % _asrt_, *SD*= 0.08), *t*(87) = 11.44, *p* < .001, *d_z_* = 1.22, CI 95% [1.01, 1.44], η_p_² = .60. Moreover, we found a facilitation effect showing that participants committed fewer errors in congruent trials (*M* = 0.06 % _asrt_, *SD* = 0.06), as compared to neutral trials (*M* = 0.10 % _asrt_, *SD* = 0.07), *t*(87) = 4.88, *p*< .001, *d_z_* = 0.52, CI 95% = [0.30, 0.73], η_p_² = .21. Finally, we found an interference effect as well: In neutral trials, participants made fewer errors (*M* = 0.10 % _asrt_, *SD* = 0.07), than in incongruent trials (*M* = 0.17 % _asrt_, *SD* = 0.08), *t*(87) = 8.88, *p* < .001, *d_z_* = 0.95, CI 95% = [0.73, 1.16], η_p_² = .48.

**Explorative Analyses.** In additional analyses, we tested whether the ratio between participants’ keyboard and their screen, the keyboard they had used, the browser they had used, their handedness, whether they used an external monitor or a laptop, and the number of repetitions in the first and the second practice block influenced the imitation-inhibition indices. None of these factors affected any of the three automatic imitation indices for the adjusted error rate percentages, *Fs* < 3.32, *ps* > .072.

**Experiment 2**

**Error rates.** In the online sample, we found the congruency effect: The participants committed fewer errors in congruent trials (*M* = 0.06 % _asrt_, *SD* = 0.06) than in incongruent trials (*M* = 0.16 % _asrt_, *SD* = 0.08), *t*(83) = 11.43, *p* < .001, *d_z_* = 1.25, CI 95% = [1.02, 1.46], η_p_² = .61. Moreover, we found a facilitation effect: The participants made fewer errors in congruent trials (*M* = 0.06 % _asrt_, *SD* = 0.06), as compared to neutral trials (*M* = 0.08 % _asrt_, *SD* = 0.07), *t*(83) = 3.37, *p* = .001, *d_z_* = 0.37, CI 95% = [0.15, 0.58], η_p_² = .12. Finally, we found an interference effect as participants made fewer errors in neutral trials (*M* = 0.08 % _asrt_, *SD* = 0.07) than in incongruent trials (*M* = 0.16 % _asrt_, *SD* = 0.08), *t*(83) = 9.61, *p* < .001, *d_z_* = 1.05, CI 95% = [0.83, 1.26], η_p_² = .53.

In the laboratory sample, we found all three imitation effects for the adjusted error rate percentages as well. The congruency effect: The participants made fewer errors in congruent trials (*M* = 0.07 % _asrt_, *SD* = 0.06), than in incongruent trials (*M* = 0.16 % _asrt_, *SD* = 0.07), *t*(97) = 10.981, *p* < .001, *d_z_* = 1.10, CI 95% = [0.90, 1.30], η_p_² = .55. The facilitation effect: The participants committed fewer errors in congruent trials (*M*= 0.07% _asrt_, *SD* = 0.06), as compared to neutral trials (*M* = 0.09 % _asrt_, *SD*=0.06), *t*(97) = 3.63, *p*< .001, *d_z_* = 0.37, CI 95% = [0.16, 0.57], η_p_² = .12. And the interference effect: The participants made fewer errors in neutral (*M* = 0.09% _asrt_, *SD* = 0.06) than in incongruent trials (*M* = 0.16 % _asrt_, *SD* = 0.07), *t*(97) = 8.42, *p* < .001, *d_z_* = 0.85, CI 95% = [0.65, 1.05], η_p_² = .42.

A direct comparison of the typical imitation-inhibition effects revealed no difference in the adjusted error rate percentages between the laboratory and online sample in terms of the congruency effect (laboratory: *M* = 0.09 % _asrt_, *SD* = 0.08; online: *M* = 0.10 % _asrt_, *SD* = 0.08), *t*(180) = 1.23, *p* = .221, *d_z_* = 0.18, CI 95% = [-0.11, 0.49], η_p_² = .03, the facilitation effect (laboratory: *M* = 0.02 % _asrt_, *SD* = 0.06; online: *M* = 0.02 %, *SD* = 0.06), *t*(180) = 0.10, *p* = .920, *d_z_* = 0.02, CI 95% = [- 0.24, 0.21], η_p_² < .001, and the interference effect (laboratory: *M* = 0,07 % _asrt_, *SD* = 0,08; online: *M* = 0,08 % _asrt_, *SD* = 0,08), *t*(180) = 1.36, *p* = .176, *d_z_* = 0.20, CI 95% = [- 0.09, 0.48], η_p_² = .04.

**Exploratory Bayes Factors for Error Rates.** In another exploratory analyses, we tested the null hypothesis that there is no meaningful difference between the automatic imitation effects of the laboratory and the online sample with the adjusted error rate percentages by applying Bayesian statistics. We calculated the BF_10_ with the default priors (Cauchy prior width *r* = 0.707) in JASP (Version 0.11.1.0; JASP Team, 2019). The BF_10_ values ranged from anecdotal evidence for the null hypothesis to substantial evidence for the null hypothesis, with a BF_10_ = 0.32 for the difference between the congruency effects, a BF_10_ = .16 for the difference between the facilitation effects, and a BF_10_ = 0.38 for the difference between the interference effects (cf. Jeffreys, 1998).

**Explorative Analyses.** Similarly, as in Experiment 1, we conducted different additional exploratory analyses. These analyses again showed that neither the ratio between keyboard and screen, the keyboard participants had used, the browser participants had used, handedness, whether they had used an external monitor or a laptop, nor the number of repetitions in the first and the second practice block influenced the imitation-inhibition indices in terms of the adjusted error rate percentages, *Fs* < 2.96, *ps* > .087.

**Experiment 3**

**Error rates.** As for the non-adjusted error rate percentages we found both the main effect for imitative compatibility, *F*(1, 92) = 14.52, *p <* .001, η_p_² = .14, and the main effect for spatial compatibility, *F*(1, 92) = 80.43, *p <* .001, η_p_² = .47. This means that participants made fewer errors in imitative congruent trials (*M* = 0.09 % _asrt_, *SD* = 0.07) than in imitative incongruent trials (*M* = 0.11 % _asrt_, *SD* = 0.07). Likewise, they made fewer errors in spatially compatible trials (*M* = 0.08 % _asrt_, *SD* = 0.05) than in spatially incompatible trials (*M* = 0.13 % _asrt_, *SD* = 0.07). The interaction was not significant, *F*(1, 92) = 0.002, *p =* .963, η_p_² < .001. Contrast analyses revealed that irrespective of spatial compatibility, participants made fewer errors in congruent trials than in incongruent trials in both the spatial congruent condition, *F*(1, 92) = 8.16, *p* = .005, η_p_² = .08, and the spatial incongruent condition, *F*(1, 92) = 9.07, *p =* .003, η_p_² = .09.

**Experiment 4**

**Error rates.** The results of the adjusted error rate percentages mirrored those of the non-adjusted ones. That is, we found the main effect for congruency, *F*(1, 103) = 47.45, *p* < .001, η_p_² = .32, meaning that participants made fewer errors in congruent trials (*M* = 0.08 % _asrt_, *SD* = 0.06) than in incongruent trials (*M* = 0.13 % _asrt_, *SD* = 0.07). We did not find a main effect for the hand condition, *F*(1, 103) = 1.11, *p* = .294, η_p_² = .01. The error rate between robotic hand trials (*M* = 0.10 % _asrt_, *SD* = 0.05) and human hand trials (*M* = 0.11 % _asrt_, *SD* = 0.06) did not differ significantly. The interaction between the congruency and the hand condition was also not significant, *F*(1, 103) = 1.60, *p* = .209, η_p_² = .02, which means that the congruency effect did not significantly vary between human (*M* = 0.05, *SD* = 0.11) and robotic hands (*M* = 0.06, *SD* = 0.10) within the adjusted error rate percentages.

**Violin Plots**

In the following we provide additional violin plots for the reaction times (see Figure 5 and 7) and the error rates (see Figure 6 and 8) of Experiment 3 and 4. This gives the possibility to get a more precise and detailed view on the data, as the violin plots show the individual mean values of the participants.

**Additional Violin Plots for Experiment 3**

**Figure 5**

*Reaction Times Imitative Compatibility x Spatial Compatibility*


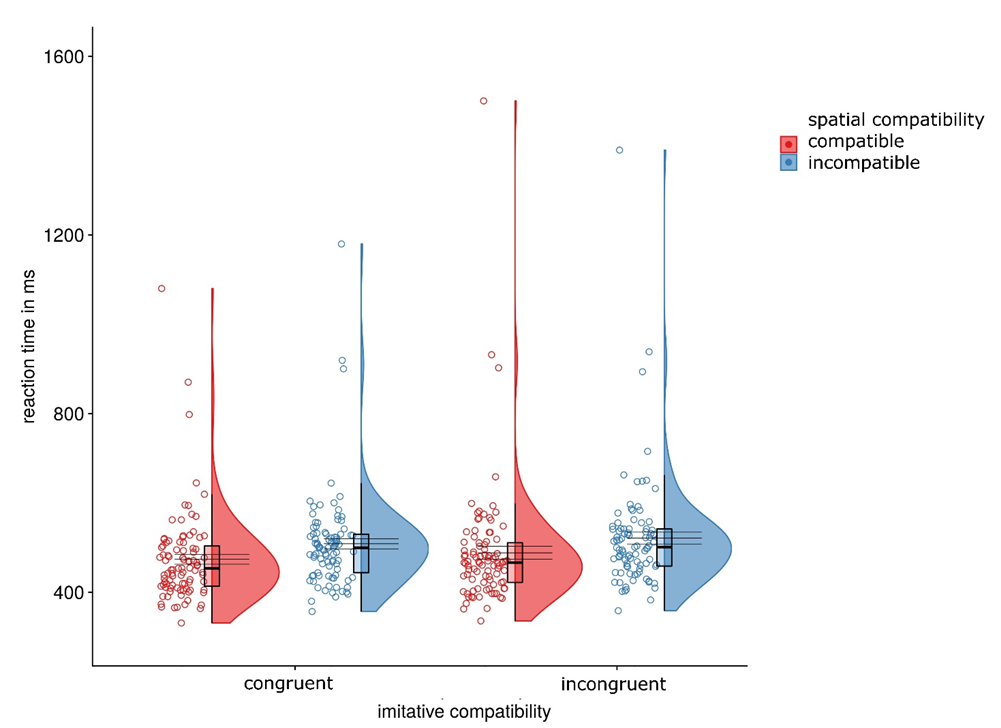


*Note.* Latencies in ms. The horizontal line represents the mean (+/- SE).

**Figure 6**

*Error Rates Imitative Compatibility x Spatial Compatibility*


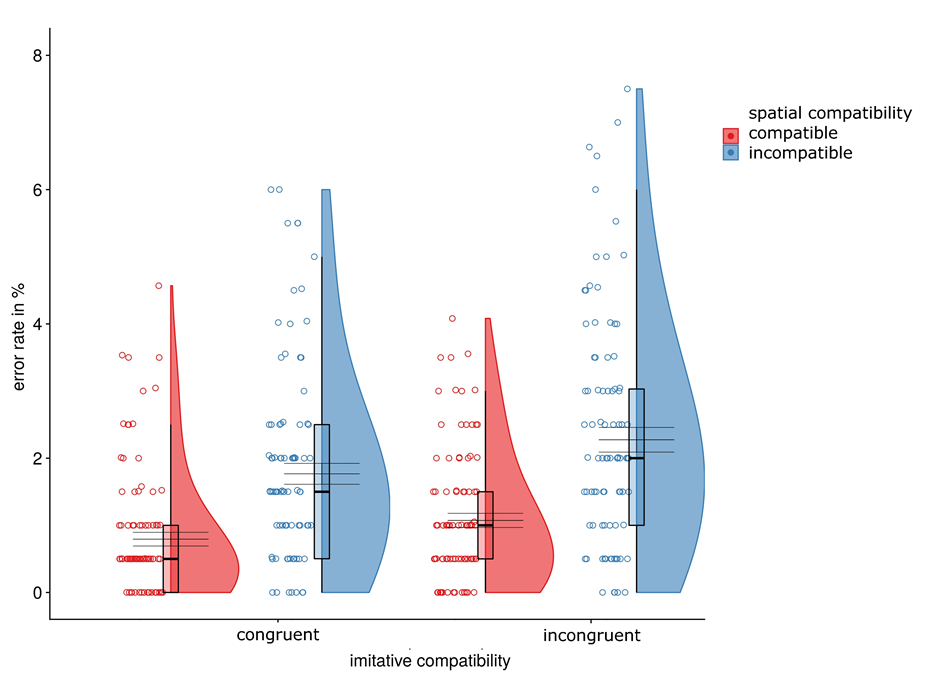


Note. Error rate percentages. The horizontal line represents the mean (+/- SE).

**Figure 7**

*The Difference in the Trial Conditions for the Latencies*


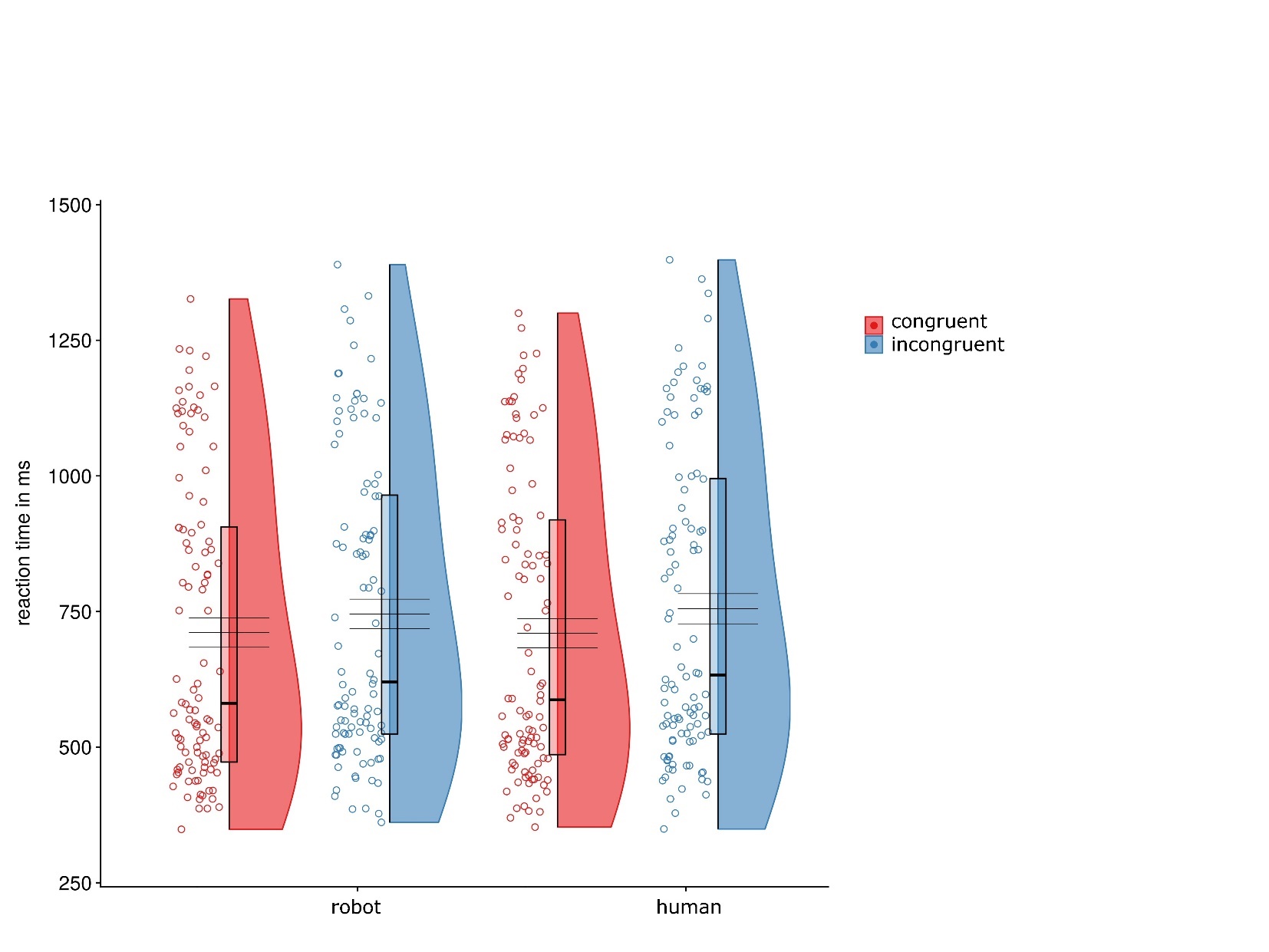


*Note.* Latencies in ms. The horizontal line represents the mean (+/- SE).

**Figure 8**

*The Difference in the Trial Conditions for the Error Rates*


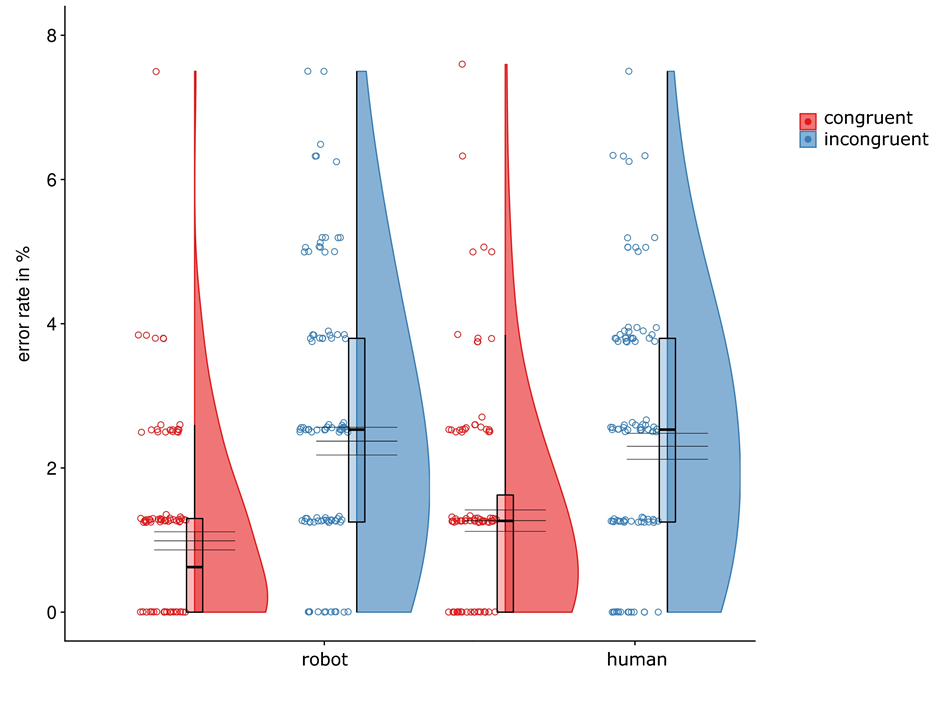


*Note.* Error rates percentages. The horizontal line represents the mean (+/- SE).

**References**

Ahrens, W. H., Cox, D. J., & Budhwar, G. (1990). Use of the arcsine and square root transformations for subjectively determined percentage data. *Weed Science*, *38*(4–5), 452–458. https://doi.org/10.1017/S0043174500056824

Borenstein, M., Hedges, L. V., Higgins, J. P. T., & Rothstein, H. R. (2010). A basic introduction to fixed-effect and random-effects models for meta-analysis. *Research Synthesis Methods*, *1*(2), 97–111. https://doi.org/10.1002/jrsm.12

Cracco, E., Bardi, L., Desmet, C., Genschow, O., Rigoni, D., De Coster, L., Radkova, I., Deschrijver, E., & Brass, M. (2018). Automatic imitation: A meta-analysis. *Psychological Bulletin*, *144*(5), 453–500. https://doi.org/10.1037/bul0000143

Dettori, J. R., Norvell, D. C., & Chapman, J. R. (2022). Fixed-Effect vs Random-Effects Models for Meta-Analysis: 3 Points to Consider. *Global Spine Journal*, *12*(7), 1624–1626. https://doi.org/10.1177/21925682221110527

Field, A. P. (2001). Meta-analysis of correlation coefficients: A Monte Carlo comparison of fixed- and random-effects methods. *Psychological Methods*, *6*(2), 161–180. https://doi.org/10.1037/1082-989x.6.2.161

R Core Team. (2022). R: A language and environment for statistical computing. *R Foundation for Statistical Computing, Vienna, Austria*. https://www.R-project.org/.

Viechtbauer, W. (2010). Conducting meta-analyses in R with the metafor package. *Journal of Statistical Software*, *36*, 1–48. https://doi.org/10.18637/jss.v036.i03
